# Antiviral mechanisms of two broad-spectrum monoclonal antibodies for rabies prophylaxis and therapy

**DOI:** 10.3389/fimmu.2023.1186063

**Published:** 2023-08-10

**Authors:** Maira Zorzan, Martina Castellan, Matteo Gasparotto, Guilherme Dias de Melo, Barbara Zecchin, Stefania Leopardi, Alex Chen, Antonio Rosato, Alessandro Angelini, Hervé Bourhy, Davide Corti, Laura Cendron, Paola De Benedictis

**Affiliations:** ^1^ Laboratory for Emerging Viral Zoonoses, FAO and National Reference Centre for Rabies, Department for Research and Innovation, Istituto Zooprofilattico Sperimentale delle Venezie, Legnaro, Italy; ^2^ Department of Biology, University of Padua, Padova, Italy; ^3^ Institut Pasteur, Université Paris Cité, Lyssavirus Epidemiology and Neuropathology Unit, WHO Collaborating Centre for Reference and Research on Rabies, Paris, France; ^4^ Vir Biotechnology, San Francisco, CA, United States; ^5^ Department of Surgery, Oncology and Gastroenterology, University of Padua, Padua, Italy; ^6^ Immunology and Molecular Oncology Diagnostics, Veneto Institute of Oncology, Padua, Italy; ^7^ Department of Molecular Sciences and Nanosystems, Ca’ Foscari University of Venice, Mestre, Italy; ^8^ European Centre for Living Technology (ECLT), Venice, Italy; ^9^ Humabs BioMed SA, a subsidiary of Vir Biotechnology, Bellinzona, Switzerland

**Keywords:** rabies virus, glycoprotein, immunotherapy, Fc-mediated effector functions, monoclonal antibodies

## Abstract

Rabies is an acute and lethal encephalomyelitis caused by lyssaviruses, among which rabies virus (RABV) is the most prevalent and important for public health. Although preventable through the post-exposure administration of rabies vaccine and immunoglobulins (RIGs), the disease is almost invariably fatal since the onset of clinical signs. Two human neutralizing monoclonal antibodies (mAbs), RVC20 and RVC58, have been shown to be effective in treating symptomatic rabies. To better understand how these mAbs work, we conducted structural modeling and *in vitro* assays to analyze their mechanisms of action, including their ability to mediate Fc-dependent effector functions. Our results indicate that both RVC20 and RVC58 recognize and lock the RABV-G protein in its pre-fusion conformation. RVC58 was shown to neutralize more potently the extra-cellular virus, while RVC20 mainly acts by reducing viral spreading from infected cells. Importantly, RVC20 was more effective in promoting effector functions compared to RVC58 and 17C7-RAB1 mAbs, the latter of which is approved for human rabies post-exposure treatment. These results provide valuable insights into the multiple mechanisms of action of RVC20 and RVC58 mAbs, offering relevant information for the development of these mAbs as treatment for human rabies.

## 1 Introduction

Rabies is an acute progressive viral encephalomyelitis, a major neglected tropical disease circulating almost worldwide (1). Canine rabies is estimated to cause 59,000 human deaths, mainly in Africa and Asia, and over 3.7 million disability-adjusted life years (DALYs) are lost every year (1, 2). As the disease is almost 100% fatal following the onset of symptoms, rabies prevention in humans is carried out primarily through post-exposure prophylaxis (PEP), which is delivered to more than 29 million exposed individuals every year (2). Standard PEP consists of accurate and timely wound washing and administration of vaccine and rabies immunoglobulins (RIGs) (1). Notably, the infiltration of RIGs into the wound (the site of possible exposure) provides immediate passive immunity overcoming the absence of pre-existing endogenous antibodies. Despite the undoubted efficacy of the available PEP protocols (1), the availability of either human or equine-origin RIGs is limited in most endemic areas where they are urgently needed. Over the years, the scientific community has attempted to develop several anti-rabies virus (RABV) monoclonal antibodies (mAbs) as alternatives to RIGs (3, 4), with three products so far available in the Indian or Chines market, namely, Rabishield (containing a unique human [h] mAb, 17C7-RAB1) (5), Twinrab (containing a cocktail of two murine mAbs, M777-16-3 and 62-71-3) (6), and Xunke (composed of a unique hmAb, ormutivimab) (7, 8). Additional products may become commercially available in the next future, such as CL184 (a cocktail of two human mAbs, CR57, and CR4098) (9), SYN023 (a cocktail of two humanized mAbs, called CTB011 and CTB012) (10), the cocktail of human mAbs 11B6 + NP-19-9 (11), and a combination of three human mAbs (CR57, RV08, and RV3A5) (12). In spite of preliminary evidence showing that mAbs might clear RABV from infected animals (13), no effective therapy for symptomatic rabies has been developed to date. Out of the 14 documented survivors of symptomatic rabies, all but one received preventive vaccine and PEP (14). The exceptional survivor with no history of vaccination had anti-RABV antibodies, suggesting an important role of the immune response in controlling viral spread (15). Based on these evidences, an immunotherapy for symptomatic rabies has been successfully attempted in a pre-clinical study (16). A cocktail including two human mAbs (RVC20 and RVC58) was able to cure early symptomatic rabies if administered alone at a high dose. More specifically, this approach was based on a brain infusion (*via* the intracerebroventricular route) of the RVC20–RVC58 cocktail combined with peripheral intramuscular injection (16). Based on previous characterization, RVC20 and RVC58 target antigenic sites I and III of the ectodomain of the RABV glycoprotein (RABV-G), respectively (17). In addition, the x-ray structure of mAb RVC20 in complex with RABV-G showed that binding of RVC20 prevents the pH-dependent G conformational rearrangements required for fusion of the viral envelope with endosomal membranes (18). Despite the x-ray structural analysis of RVC58 binding to RABV-G is not available yet, recent Surface Plasmon Resonance (SPR) analysis shows that both mAbs bind the RABV-G at pH 7.5 (19), indicating they both recognize the protein in its pre-fusion conformation, and that, similarly to what has previously been described for RVC20 (18), RVC58 blocks the pre-fusion conformation of the RABV-G. Nevertheless, despite the progress toward the fine characterization of the RABV-G structure and the concomitant structure analysis and dissection of the receptor binding of different mAbs (18–21), the biological effects of these mAbs at cellular level is poorly understood. Similarly, the ability of these mAbs to mediate effector functions has been investigated in mice administered with the Fc-mutated version of RVC20 and RVC58 mAbs [LALA Fc unable to bind to Fc gamma receptors (FcγRs)], indicating a potential role of Fc-dependent mechanisms at controlling disease progression (16). Given the limited response of the host immune system to rabies infection (22, 23) and the minimal cell damage on RABV-infected neuronal cells expected from a successful treatment (16), it will be crucial to further study the role of effector functions promoted by mAbs used for both prophylaxis and therapy in order to better understand their role for viral clearance *in vivo*.

In the present study, we investigated the neutralizing, binding, and Fc-mediated functions of RVC20 and RVC58, in order to elucidate their *in vitro* mechanisms of action. The neutralizing capability of these mAbs against RABV was tested under varying conditions of viral replication to simulate early and delayed mAbs therapy following RABV exposure. Structural modeling was used to gain insights on the paratope-epitope affinities and mechanistic effects and bioassays were also performed to evaluate complement activation and engagement of human Fcγ receptors by the Fc portion of RVC20 and RVC58 bound to RABV-infected target cells.

## 2 Materials and methods

### 2.1 Cell lines and RABV strains

All cells were cultured at 37°C, 5% CO_2_. Human neuroblastoma SH-SY5Y (ATCC^®^ cat. n. CRL-2266) and SK-N-SH (ATCC^®^ cat. n. HTB-11^™^) cells were grown in Dulbecco's Modified Eagle Medium (DMEM) supplemented with 10% heat-inactivated Fetal Calf Serum (FCS) at 37°C and 5% CO_2_. RABVs used for the study were alternatively (i) the Challenge Virus Standard (CVS-11; ATCC^®^ cat. n. VR-959) RABV strain or (ii) the recombinant RABV expressing the GFP (Tha-GFP) (24) based on the wild isolate Thailand RABV (isolate 8743 THA, EVAg collection, Ref-SKU: 014 V-02106).

### 2.2 *In vitro* investigation of the mechanisms of action of the RVC20-RVC58 cocktail

**Figure 1:**
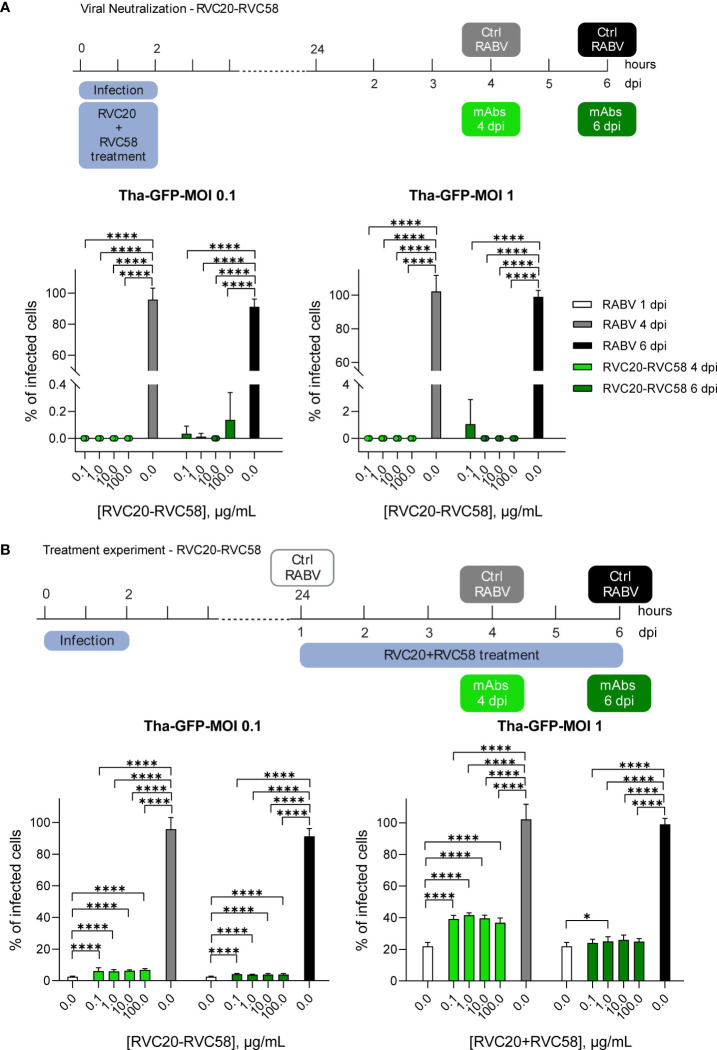
RVC20-RVC58 mAbs cocktail ability to neutralize and treat RABV infection. **(A)** RVC20-RVC58 cocktail neutralization of the recombinant RABV (Tha-GFP) in SK-N-SH cells. The mAbs cocktail was added along with the viral inoculum; the cocktail-RABV was then removed after 2h. Cells were fixed at 4 or 6 dpi. **(B)** RVC20-RVC58 mAbs cocktail-mediated inhibition of Tha-GFP spread to SK-N-SH infected cells. 1 dpi, the mAbs cocktail was added to the cells up to 4 or 6 dpi. Bars represent mean ± SD (*n* = 3 independent experiments). Unpaired t-tests of the RABV control cells against each concentration of the mAbs cocktail under investigation (4 or 6 dpi, respectively) and of each mAbs concentration against the others. Only statistically significant comparisons are shown (* = p < 0.05; **** = p < 0.0001). Raw cell counts are available in **Supplementary Tables S1–S5**. Cartoons created with BioRender.com (Agreement number JD251EVLE9 and ZJ251EVMO5).

For the results shown in **Figure 1**, SK-N-SH cells were seeded at a density of 1.6 × 10^4^ cells/cm^2^ in 96-well plates (Cat. n. 655086, Greiner Bio, Kremsmünster, Austria). Twenty-four hours after seeding, the SK-N-SH cells were incubated for 2h with the desired multiplicity of infection (MOI) of the RABV Tha-GFP; subsequently, the viral suspension was removed and fresh culture medium was added. An equal amount of RVC20 and RVC58 (0.1 + 0.1, 1 + 1, 10 + 10, and 100 + 100 µg/mL) was added as a cocktail either during the infection (neutralization; 2h treatment) or after the infection [treatment; from day 1 to 4 or 6 days post-infection (dpi)]. Cells were fixed at different dpi and further processed as described in **Supplementary Materials**.

### 2.3 *In vitro* comparison of the neutralizing activity and the inhibition of viral spread of the RVC20 and RVC58 mAbs

SH-SY5Y cells were seeded at a density of 1.8 × 10^5^ cells/mL on round glass slides placed in 24-well plates (Falcon) with or without RABV CVS-11 (MOI 0.1), for the treatment or neutralization experiment, respectively.

**Figure 2:**
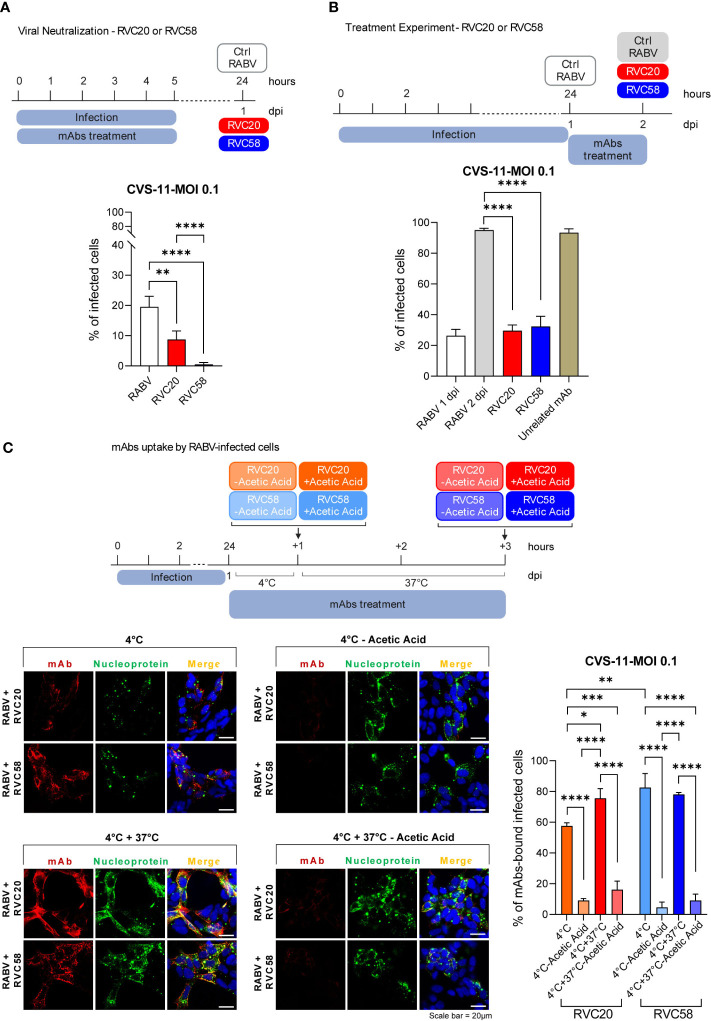
RVC20 and RVC58 ability to neutralize and treat RABV infection. **(A)** Single antibody neutralization in SH-SY5Y cells. RVC20 or RVC58 (0.6 µg/mL) was pre-incubated with RABV (CVS-11) (MOI 0.1) for 1h at 37°C. The mAb-RABV mix was then added to the cells and removed after 5h. Cells were fixed at 1 dpi. **(B)** Single antibody-mediated inhibition of viral spread in SH-SY5Y cells. 1 dpi with RABV (CVS-11) at MOI 0.1, RVC20 or RVC58 (0.6 µg/mL) was added to the cells for 24h. Cells were fixed 2 dpi. Bars represent mean ± SD (*n* = 2 independent experiments). Unpaired t-tests of RABV-infected cells against RVC20 or RVC58, and RVC20 against RVC58. Only statistically significant comparisons are shown (** = *p* < 0.01; **** = *p* < 0.0001). Cartoons created with BioRender.com (Agreement number XG251EXJXJ and NB251KBBNZ). **(C)** Immunofluorescence images (left) of SH-SY5Y cells infected with RABV (CVS-11) (MOI 0.1) and incubated with RVC20 or RVC58 at 4°C/4° + 37°C, with or without acetic acid treatment. Red: RVC20 or RVC58 monoclonal antibody; green: viral nucleoprotein; blue: DAPI (nuclear staining). Scale bar = 20 µm. The percentage of mAbs-bound RABV-infected cells (right) is also shown. Bars represent mean ± SD (*n* = 2 independent experiments). Only statistically significant results are shown (* = *p* < 0.05; ** = *p* < 0.01; *** = *p* < 0.001; **** = *p* < 0.0001). Cartoons created with BioRender.com (Agreement number VD25HUWSIM).

To investigate the capability of each single mAb to neutralize extracellular virions (**Figure 2A**), cells were infected 24h after seeding, with a mix of CVS-11 and either RVC20 or RVC58 (0.6 µg/mL) previously incubated for 1h at 37°C. The selected mAbs concentration represents the average dose likely distributed in the central nervous system of experimentally treated Syrian hamsters (**Supplementary Figure S1**). Five hours after the infection, the viral and mAb inoculum was removed and fresh medium added. Cells were fixed 1 dpi and processed as described in **Supplementary Materials**.

For the results of the treatment experiment reported in **Figure 2B**, RABV CVS-11 inoculum was washed with fresh medium 1 dpi and cells were treated with either RVC20, RVC58, or an unrelated mAb [rIgG1 ZKA78 (25)] at a concentration of 0.6 µg/mL. Cells were fixed at 2 dpi and processed as described in **Supplementary Materials**.

### 2.4 Determination of RVC20 and RVC58 internalization rate by RABV-infected cells

SH-SY5Y cells were seeded and infected with RABV CVS-11 (MOI 0.1) as described in paragraph 2.3. Cells were treated 1 dpi either with RVC20 or RVC58 (0.6 µg/mL) and kept at 4°C for 1h. Cells were then washed with cold complete medium and fixed or further incubated at 37°C for 2h in order to allow mAbs’ internalization. After incubation, the cells were fixed or treated before fixation with acetic acid (0.2M; pH 2.5) for 30 s, in order to remove the antibodies bound to the cell surface (**Figure 2C**). Fixation and further processing are described in the **Supplementary Materials**.

### 2.5 Complement-dependent-cytotoxicity assay

Activation of complement-dependent cytotoxicity (CDC) was evaluated by an *in vitro* colorimetric cytotoxicity assay. Briefly, SH-SY5Y cells were infected with RABV CVS-11 (MOI 0.1) and plated in 96-well plates at 3.0 × 10^4^ cells per well. Properly diluted mAbs or Fab forms of mAbs were added to cells 2 dpi and plates were incubated 20 min at room temperature. Guinea pig complement (Cat. n. CSI053022, Sclavo Diagnostics, Siena, Italy) was reconstituted with 1 mL DMEM, diluted 1:30 in DMEM and finally added (50 µl/well) to antibody-treated cells. After a 3h incubation at 37°C, cell viability was measured using AlamarBlue^®^ HS-cell viability reagent (Cat. n. DAL105, Thermo Fisher Scientific, Massachusetts, USA), according to the manufacturer’s instructions. Data were normalized to minimal cell lysis, represented by untreated infected cells incubated with guinea pig complement.

### 2.6 Antibody-dependent cell cytotoxicity and antibody-dependent cell phagocytosis assays

Induction of antibody-dependent cell cytotoxicity (ADCC) and antibody-dependent cell phagocytosis (ADCP) by RVC20 and RVC58 mAbs were evaluated in RABV CVS-11–infected SH-SY5Y cells using two commercially available kits. The bioassays are bioluminescent reporter assays exploiting effector cells engineered for the expression of human FcγRs involved in mAb Fc portion binding and signal transduction for the promotion of the effector function. Briefly, SH-SY5Y cells were infected with RABV CVS-11 MOI > 10 to maximize the amount of the G protein expressed at the cell surface and plated in white 96-well plates at 3.0 × 10^4^ cells/well. Threefold serially diluted mAbs at an initial concentration of 40 µg/mL were added 18h to 24h later and plates were incubated for some minutes at room temperature to allow mAbs to bind to RABV-infected target cells. Engineered effector cells were thawed according to the manufacturer’s instructions (ADCC Reporter Bioassay—FcγRIIIa receptor—cat. n. G7501 and FcγRIIa-H ADCP Reporter Bioassay cat. n. G9901 Promega, Fitchburg, Wisconsin, USA) and then added to assay plates at a 2.5:1 effector-to-target-cell ratio. After 6h of incubation at 37°C, activation of specific effector functions was quantified using Bio-Glo Luciferase Assay System (Promega) and a GloMax-Multi+ Detection System. Measured relative luciferase units (RLUs) were converted into Fold of Induction [luminescence (induced–background)/luminescence (no antibody control–background] and plotted against the logarithm of the antibody concentrations.

### 2.7 Molecular modeling

Models of the extravirion domain of RABV-G strain CVS-11 were obtained by homology modeling at the swissmodel webserver (26, 27). The crystal structures of RABV-G at basic pH (6LGX) and acid pH (6LGW) were used as templates to model RABV-G in the pre- and post-fusion conformation, respectively. The final models included residues spanning from Lys1 to Ser392 and their quality evaluated using GQME and QMEAN6 indexes. RVC58 was modeled *via* Rosetta online server (28) and docked to RABV-G in pre- and post-fusion conformation using HADDOCK 2.4 webserver (29, 30). Further information can be found in **Supplementary Materials**. Protein complexes interaction surface and contacts were analyzed by PISA server (PDBe PISA v1.52). ΔG_bind_ between antibodies and RABV-G were determined by umbrella sampling. Analogous steered molecular dynamics protocols have been recently applied to calculate potential of mean force (PMF) and estimate ΔG_bind_, as described in (31–35). All the details about umbrella sampling protocol can be found in **Supplementary Materials**.

### 2.8 Statistical analysis

**Figure 3:**
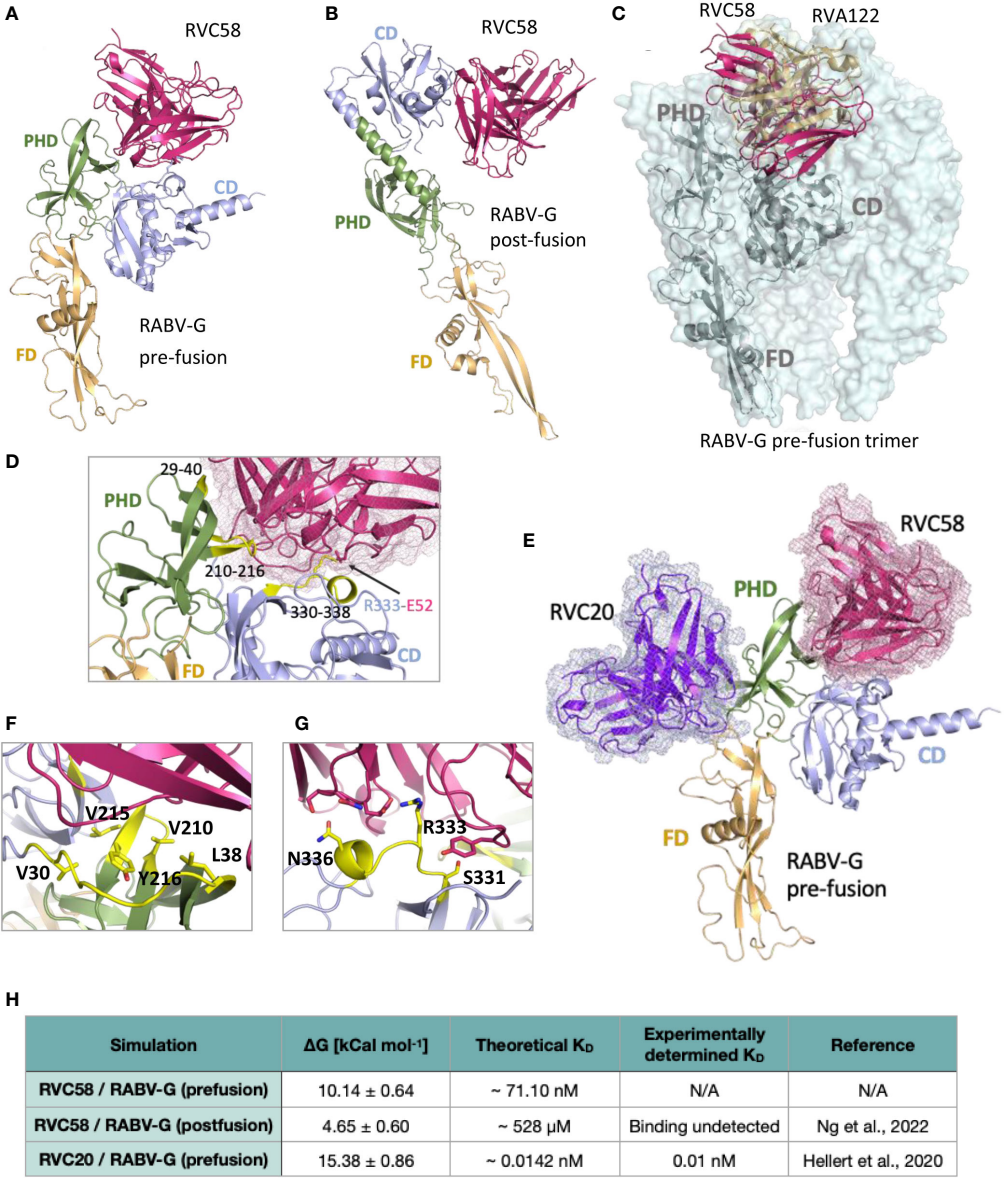
Analysis of the RVC58 binding to RABV-G. Binding poses of RVC58 (magenta) to RABV-G (teal) in pre-fusion **(A)** and post-fusion **(B)** conformations. RABV-G glycoprotein has been colored as follows, to distinguish the three domains: FD domain in light orange, PHD domain in smudge and CD domain in light blue, respectively. **(C)** Superposition of RVC58 model (magenta) in complex with RABV-G (gray) and the CryoEM structure of RVA122 (sand) in complex with the pre-fusion trimer of RABV-G (palecyan surface; PDB:7U9G). Superposition of RVA122/RVC58:RABV-G complexes has been limited to a single chain for clarity. **(D, F, G)** Zoomed view of the contact region of RVC58 in complex with RABV-G in the pre-fusion state. The RABV-G epitopes directly involved in the interaction have been highlighted in yellow. **(E)** Superposition of RVC58 model (magenta) in complex with RABV-G (light range, smudge, and light blue) and RVC20 structure [purple (18);]. **(H)** ΔG_bind_ and theoretical *K_D_* of the complexes simulated in this work compared with available data.

**Figure 4:**
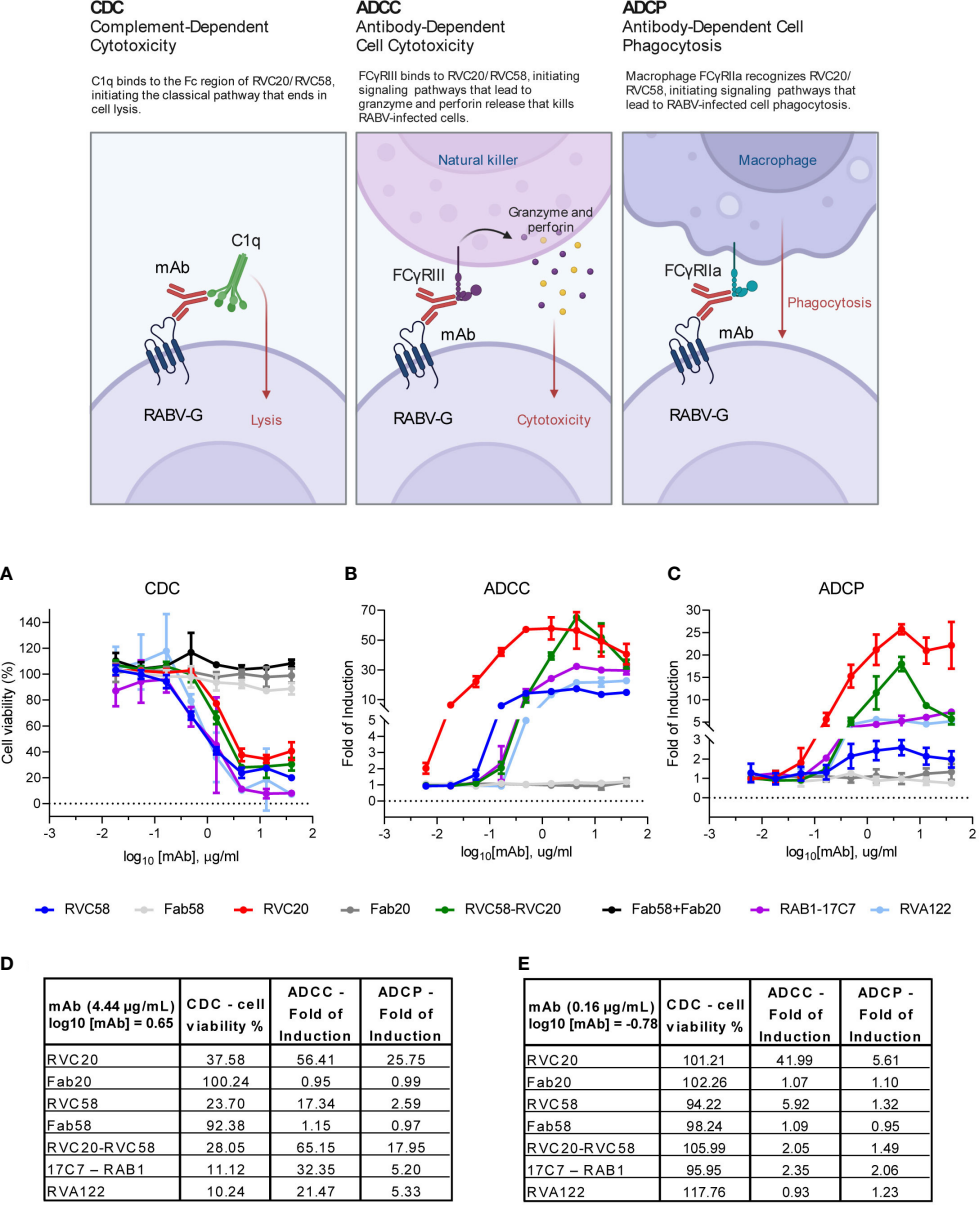
RVC20 and RVC58 promote immune effector functions in RABV-infected cells. RVC20, RVC58, RVC20-RVC58 cocktail, Fab58, Fab20, Fab20-Fab58 cocktail, RVA122 and the commercially available 17C7-RAB1 were evaluated. **(A)** In-house assay for the induction of complement-dependent cytotoxicity (CDC); we evaluated cell viability percentage (Alamarblue staining) after SH-SY5Y cells treatment 1 dpi with scalar doses of antibodies. Promotion of antibody-dependent cell cytotoxicity (ADCC) **(B)** and antibody-dependent cell phagocytosis (ADCP) **(C)** *via* human FcγRIII and FcγRIIa receptors, respectively, analyzed through a commercially available (Promega) bioluminescent reporter assays, 30h and 24h post-infection, respectively. Results are presented as Fold of Induction for every mAb dilution. Mean ± SD of every mAb concentration tested is represented (*n* = 2 independent experiments). Cartoon created with BioRender.com (Agreement number YI24XFS6KG). **(D)** Percentage of cell viability (CDC) and Fold of Induction (ADCC and ADCP) referring to the experiments displayed in **(A–C)**; values are expressed as mean results obtained at 4.44 µg/mL (or log_10_ [mAb] = 0.65) for each tested mAb or for the cocktail. See **Supplementary Table S7** for statistic details. **(E)** Percentage of cell viability (CDC) and Fold of Induction (ADCC and ADCP) referring to the experiments displayed in **(A–C)**; values are expressed as mean results obtained at 0.16 µg/mL (or log10 [mAb] = -0.78) for each tested mAb or for the cocktail. See **Supplementary Table S8** for statistic details.

All statistical analyses were performed using GraphPad Prism 9. For the *p*-values of the results presented in **Figures 1**, **2** and **Supplementary Figure S2A**, we applied an unpaired t-test. We did not apply any statistical analysis of the data presented in **Figure 3**. For the analyses described in **Figure 4** (**Supplementary Tables S7, S8**), we applied a one-way ANOVA with Tukey’s multiple comparisons. For the statistical analyses described in **Figure 2C**, we applied a two-way ANOVA with Šídák’s multiple comparisons. For all statistics, we considered a *p*-value < 0.05 as significant.

## 3 Results

### 3.1 RVC20-RVC58 cocktail neutralizes extracellular RABV and inhibits viral spread from infected cells

To investigate the interaction between the virus and the mAbs before infection (i.e., neutralization of the virions), RABV at two different MOIs was pre-incubated with increasing concentration of the RVC20-RVC58 cocktail. The mAbs cocktail displayed a significant neutralizing effect under all tested conditions, confirming its potency in preventing RABV entry into the cells (**Figure 1A** and **Supplementary Table S1**).

Next, we investigated the ability of mAbs to inhibit viral spread from infected cells by adding the mAbs cocktail after RABV infection. A significant reduction in the viral spread was observed in cells treated with the cocktail compared with control cells fixed at the same time post-infection. Notably, no differences were observed between the mAbs doses used and the observed inhibitory effect. On the contrary, the cocktail displayed greater efficacy when cells were challenged with a lower viral dose (i.e., MOI 0.1 vs. MOI 1).

As expected, the rate of infection before the treatment (1 dpi) was significantly lower than what observed at 4 or 6 dpi (meaning 3 or 5 days treatment), with the only exception of the comparisons of MOI 1 experiments at 6 dpi. Without mAbs treatment, we observed that the infected cells were almost 100% at 4 dpi, regardless of the specific MOI used. Treatment with mAbs, however, contained the infection progression despite the mAbs concentration, with an increase rate varying only from 1.1 to 2.7 times, which is equivalent to (i) 6.3 and 3.9% of infected cells, respectively, at 4 and 6 dpi at MOI 0.1 and (ii) 39.2 and 25% of infected cells at 4 and 6 dpi at MOI 1, respectively (**Figure 1B** and **Supplementary Table S1**). Notably, the cell counts indicated a reduced number of cells in the infected, untreated group when compared with both the infected and treated group, as well as the uninfected, untreated group (**Supplementary Tables S2–S5**). These differences were more evident at 6 dpi at MOI 1, likely indicating a reduction in the replication activity of the infected cells, which might also explain the differences observed between 1 and 6 dpi in terms of treatment’s efficacy.

### 3.2 RVC20 and RVC58 neutralize the extracellular virus and inhibit an early and a late phase of the viral life cycle

We then investigated the effect of each mAb in neutralizing extracellular virions or in reducing viral spread. When added to the viral inoculum, both antibodies were able to significantly neutralize RABV, although RVC58 displayed a greater neutralizing potency compared with RVC20 (**Figure 2A**), in agreement with previous results (16). When administered post-infection, RVC20 and RVC58 were not able to significantly reduce RABV infection rate but acted by blocking viral spread (**Figure 2B**). Notably, no statistically significant differences in the RVC20 and RVC58 efficacies were observed in the treatment experiment in the range of mAbs concentration used. Similarly to what observed for the mAbs cocktail, the infected and treated cells displayed viability similar to the unifected and non-treated cells; on the contrary, the RABV-infected and non-treated cells displayed a loss of cell viability that became statistically significant 4 dpi (**Supplementary Figure S2A**).

In order to evaluate the actual uptake of the mAbs by the RABV-infected cells, we assessed the amount of the internalized RVC20 and RVC58 according to consolidated protocols (13, 36–39). When incubated for 1h at 4°C, mAbs bind their specific RABV-G epitopes on the cell surface. Treatment of these cells with a solution of acetic acid led to an almost complete removal of the RVC20 and RVC58, indicating that at 4°C, antibodies are not internalized and are located at the cell surface (**Figure 2C**). The same pattern was also observed when cells were shifted from 4°C to 37°C (promoting endocytosis); of note, we did not observe a significant difference in the number of mAbs-bound RABV-infected cells at 4°C or at 4°C + 37°C after acid treatment, indicating no internalization of both antibodies (**Figure 2C**). Consistently, we observed that RVC20 and RVC58 appeared to be mainly located at the cell membrane of infected cells, as shown by antibody co-localization with the cell membrane marker fluorescent wheat germ agglutinins (WGA), (**Supplementary Figure S2B** and **Supplementary Videos S1–S6**). In this context, it should be noted that WGA was used prior to cell permeabilization (**Supplementary Materials and Methods**), allowing the specific staining of the external cell membranes.

### 3.3 Molecular modeling predicted the high affinity binding to the RABV-G pre-fusion conformation by RVC58

To provide a structural basis for RVC58 binding and activities, we superimposed the crystal structure of RVC20 in complex with domain III-fragment of the RABV-G protein (18) to that of the docked model of RABV-G ectodomain in complex with RVC58. A pose for RVC58 binding to RABV-G was obtained by constraining docking search to the epitope mapping data available. Molecular modeling revealed that RVC58 binds to pre-fusion RABV-G defining an extended interaction surface of 1336 Å^2^, spanning both the pleckstrin homology domain (PHD) and the central domain (CD; domains (**Figures 3A, D**). While binding to CD mostly involves residues included or close to antigenic site III, the binding region in the PHD defines a second and a third epitope, spanning between residues 210–216 (**Figures 3A, D**) and 29–40, respectively. Interestingly, epitope 210–216 is part of the interaction surface of trimeric pre-fusion RABV-G in complex with neutralizing mAb RVA122 (PDB 7U9G, **Figure 3C**) (20). This antibody contacts antigenic site III in a pose very similar to that of RVC58 and buries RABV-G epitope 210–216 (**Figure 3E**).

Modeling suggested that binding of RVC58 to antigenic site III is driven by a salt bridge between Glu52 of RVC58 and Arg333 of RABV-G, which is further stabilized by polar bonds throughout residues Ser331 and Asn336 of antigenic site III (**Figures 3A, D, F** and **Supplementary Figures S3A–C**). Binding to the PHD epitope contributes to burying a large glycoprotein surface and favorite multiple hydrophobic interactions, mainly defined by RABV-G Val210, Leu215, Tyr216, Val29, Val30, and Leu38 (**Figure 3G** and **Supplementary Figure S3A**) and further sustained by hydrogen bonds with main chain of PHD epitopes. These results agree with previous studies indicating that RVC58 binds to the antigenic site III region (330–338), most probably engaging an epitope partially overlapping with the one recognized by the well-characterized CR4098 antibody (17). Notably, RVC58 predicted binding region also partially overlaps with that of another previously characterized mAb (523–11), while engaging a contact surface more similar to the one covered by RVA122 (**Figure 3D**). Indeed, binding of both RVC58 and RVA122 to PHD domain may contribute to explain why RVC58 is able to lock RABV-G in the pre-fusion conformation, inhibiting the conformational transition to the post-fusion state (19, 20).

Analysis of the docked pose of RVC58 to post-fusion RABV-G displays a much smaller interaction surface (930 Å^2^) (**Figure 3B** and **Supplementary Figures 3D–F**). Antigenic site III is the only region bound in this conformation and the inter-protein contacts are predicted to be largely different. While interactions between CDRs and residues 334–336 are predicted to be conserved, the Glu52–Arg333 salt bridge appears to be lost (**Supplementary Figure S3D**). Overall, such pose suggests a lower affinity of RVC58 for RABV-G in the post-fusion conformation compared with the pre-fusion state.

To test this hypothesis, we performed umbrella sampling simulations to infer ΔG_bind_ between RVC58 and RABV-G in both pre- and post-fusion conformations. To validate our approach and compare predicted binding affinity (*K*_D_) values with those determined experimentally, we also ran simulations on the available crystal structure of the RVC20 bound to RABV-G domain III (PDB ID 6TOU) in the pre-fusion conformation (**Figure 3E**). Indeed, RVC20 sampling protocol produced *K*_D_ in the picomolar range in agreement with previously reported experimental values (**Figure 3H** and **Supplementary Figures 3G–H**) (18). Pulling simulations of RVC58 from RABV-G in the pre- and post-fusion conformations resulted in similar force versus time graphs (**Supplementary Figures S3B, E**). Moreover, ΔG_bind_ of RVC58 to RABV-G in the post-fusion conformation was found to be 4.65 ± 0.60 kcal mol-1, which corresponds to a neglible binding affinity. Conversely, we predict ΔG_bind_ to be 10.14 ± 0.64 kcal mol-1 for RVC58/RABV-G pre-fusion complex, which corresponds to a *K*_D_ in the nanomolar range (**Figure 3H**). The extent and nature of interactions explored by RVC58 through docked models strongly support the calculated nanomolar affinity toward pre-fusion RABV-G.

### 3.4 RVC20 and RVC58 mediate efficient complement-dependent cytotoxicity but RVC20 activates cell-mediated effector functions more potently than RVC58

To assess Fc-dependent effector mechanisms, RVC20 and RVC58, their relative Fab forms as well as the mAbs cocktail were evaluated. In addition, we tested RVA122, a previously characterized anti RABV-G mAb (17, 20), and 17C7-RAB1, currently marketed as Rabishield (4, 5, 40, 41). All the antibodies were tested for their ability to neutralize RABV, confirming that also Fabs retained *in vitro* neutralizing activity, to a certain extent (**Supplementary Table S6**).

Dose-dependent cytotoxicity due to the activation of the complement cascade (CDC) was induced by all the tested antibodies but the Fabs, indicating that complement activation was mediated by the Fc portion of antibodies-coated RABV-infected cells (**Figure 4A**). Of note, RVA122 and 17C7-RAB1 induced CDC to levels higher than those observed with RVC20, RVC58, or their cocktail. RVC20 was the least efficient in promoting CDC (**Figures 4A, D, E**, and **Supplementary Tables S7, S8**).

To evaluate the ability of RVC20 and RVC58 to promote ADCC and ADCP, we performed some tests to verify whether mAbs could bind and activate Fcγ receptors expressed at the surface of Jurkat cells. In contrast to the observations made for CDC, RVC20 displayed a significantly higher engagement of both human FcγRIII and FcγRIIa (mediating ADCC and ADCP, respectively) compared with all tested antibodies. RVC58, 17C7-RAB1, and RVA122 induced only modest or negligible ADCC/ADCP. Of note, at a high concentration (4.44 µg/mL), RVC58 had the lower capability to promote both ADCC and ADCP than the other tested mAbs, although it better activated ADCC compared with RVA122 at a lower concentration (0.16 µg/mL). As expected, the Fabs were not able to activate Fcγ receptors. Moreover, the glycoprofiles of RVC20 and RVC58 were found to exhibit a high degree of comparability, thereby eliminating the potential for a confounding impact on effector functions resulting from the differential composition of Fc-bound glycans (**Supplementary Table S9**).

Interestingly, the RVC20-RVC58 cocktail promoted the effector functions in a way comparable with the one of the single RVC20 at a half dosage (**Figures 4B–E** and **Supplementary Tables S7, S8**).

## 4 Discussion

It is widely recognized that antibodies can neutralize viruses through multiple mechanisms (42). Antibodies can inhibit infectivity by binding to viral particles and preventing them from binding to the host target cells (pre-attachment neutralization) (43–46). Other antibodies can interfere with the attachment of the pathogen to its target (39, 47, 48). Post-attachment neutralization mechanisms have also been described, including inhibition of fusion steps required for viral replication (39, 48–52), as hypothesized for RVC20 (18) and, more recently, for RVC58 (19), and as shown for other viral antibodies, such as anti-HA stem mAbs (53). Inhibition of later steps of the viral lifecycle also includes the Fc-dependent internalization of antibody-coated virions contributing to dampen viral replication (13, 42, 54). In addition, some antibodies can interfere with virion assembly and budding, thus affecting virus release from the infected cells (55–58). Finally, other antibody functions dependent on effector cells or effector molecules, such as the complement, have been described and can contribute to *in vivo* protection (42). In this complicated scenario, the elucidation of the mechanisms of action of mAbs requires a comprehensive approach, incorporating both *in vitro* and *in vivo* investigations.

This study describes essential information to understand the potential application of RVC20-RVC58 cocktail in either the prevention (17) or, possibly, the therapy of rabies (16), obtaining information about (i) the mAbs’ ability to neutralize the virus before its entry into the cells (**Figures 1A**, **2A**) and (ii) their ability to slow down (almost block) the viral replication in the infected cells and viral spread (**Figures 1B**, **2B** and **Supplementary Figure S2B**), as well as (iii) the activation of the effector functions (**Figure 4**). In this context, as the mAbs’ cocktail at a high dose has been previously shown to be able to cure symptomatic rabies and to finally clear the virus from the central nervous system of the infected animals (16), we deliberately split the experiments *in vitro* in order to gain different information (i.e., those related to mAb’s binding to the RABV-G or to the interaction between Fc and Fcγ receptors). Of note, RVC20 and RVC58 were tested at their limiting activity in order to assess (i) a possible dilution effect of the cocktail (**Figure 1A**) and (ii) differences between the mAbs (**Figure 2A**). Noteworthy, the experiments described in **Figures 1**, **2** were undertaken independently by two research groups, making the conclusions more consistent. We eventually corroborated empirical data by modeling the RVC58 binding to the G protein for the first time ever (**Figure 3**). Indeed, our model on RVC58 binding is supported by previous data, including the absence of antigen-binding competition between RVC20 and RVC58 (17, 19), and was also validated by superimposing the structural reconstructions of both RVC20 (18) and RVA122 (20).

Our results indicate that RVC20 and RVC58 *in vitro* can efficiently reduce RABV spreading, and strongly support an early extracellular neutralizing effect of both antibodies (**Figures 1A**, **2A**). In part, this can be attributed to their ability to prevent the fusion of the viral envelope with endosomal membranes, effectively locking RABV-G in the pre-fusion state, as previously demonstrated for RVC20 (18), later supported for both antibodies (19) and here further confirmed by the molecular dynamics simulations provided in the present study. However, we cannot exclude a pre-attachment neutralization, a mechanism that could better explain the high neutralizing activity of RVC58 (17). A significant reduction of cell-to-cell spread of infection was observed for both antibodies, suggesting that they might also have an inhibitory effect on the late phases of the viral lifecycle, such as virus assembly or budding.

Notably, the mAbs cocktail, although not completely effective, was able to restrict the infection spread *in vitro* in the treatment experiment (**Figure 1B**), where both antibodies performed equally when tested separately (**Figure 2B**). Coupled with the data on binding affinity determined through molecular dynamics simulations, these results suggest that binding of RVC58 to the RABV-G, though weaker than RVC20, might likely determine a more efficient inhibition of viral attachment. Indeed, findings from our simulations indicate that RVC58 binds to Arg333 of the RABV-G through a salt bridge, similarly to what observed for 523-11 (21) and 17C7-RAB1 antibodies (19). Notably, the residue Arg333 is known to interact with the p75NTR receptor (59) and to play a paramount role in RABV pathogenicity and neuroinvasiveness (60), thus likely explaining the great efficiency in neutralizing extracellular RABV virions observed for RVC58. In line with our results, the RABV strains bearing Arg333 to Lys mutation, known to maintain pathogenicity (61), would likely conserve the salt bridge with RVC58 observed in our simulations, while other residues such as Glu or hydrophobic and slight polar residues such as Gly, Leu, Ile, Met, Gln, Cys, or Ser, found in avirulent and unstable RABV strains (60, 62–65), might abolish the predicted interaction.

Conversely, a higher affinity binding of RVC20 for RABV-G in its pre-fusion conformation may lead to an effective inhibition of budding. This hypothesis remains to be confirmed, although it is supported by the observation of the peculiar basket-like conformation of the RNP complexes in RABV-infected cells treated with RVC20, which contrasts with the “pull” function of the RABV-G (66) (**Supplementary Figure S2B**). Notably and according to the current rhabdovirus model, RABV budding is mediated by both the viral M protein promoting evagination of the membrane with a “push” function from the inside of the cell (67, 68) and by the viral G protein exerting a “pull” effect from the outside of the membrane, where it accumulates in sites favorable for budding (G microdomains) (66). Therefore, the binding of the RVC20 antibody to RABV-G might result in the inhibition of budding *via* a mechanism that is analogous to the inhibition of membrane fusion (18). This is possibly related to the ability of RVC20 to lock the membrane-proximal region of the G protein, which is necessary for modifying membrane topology in both processes.

The results of our molecular dynamics simulations are in agreement with recently published data (19), offering the structural rationale for RVC58 behavior (**Figure 3**). Indeed, Ng and collaborators proved that RVC58, similarly to RVC20, is specific for the pre-fusion state and prevents from the conformational transition to the post-fusion one, which is bound with negligible affinity by RVC58. Failure of both mAbs in binding the RABV-G protein in its post-fusion conformation might partly explain the absence of full clearance activity of the mAb cocktail *in vitro* observed in the present study, thus suggesting that other mechanisms of action might be involved in the efficacy of such a cocktail *in vivo* (16).

Our results on Fc-mediated effector functions indicate that RVC20 is more effective than RVC58 at mediating ADCP and ADCC and that these same functions appear to be mainly mediated by RVC20 when they are in an equimolar cocktail. As both mAbs display similar gycoprofiles and remain at the cell surface without being uptaken by the infected cells, the differences observed in terms of mAb-mediated effector functions may be explained by the recognition of distinct and distal epitopes between RVC20 and RVC58. This likely results into distinct orientations and positioning of the mAb Fc fragment relative to the Fc receptors, as well as into different degrees of occupancy in the context of the binding to the trimeric RABV-G protein (i.e., whether steric clashing may prevent multiple IgG molecules to bind to neighboring monomers on the same trimer) (69, 70). Our results are consistent with the overall epitope dependence of Fc mediated effector functions of several antiviral mAbs (71, 72) (**Figure 3E**). Of note, RVC58 reaches a higher activity in terms of ADCC activation at a lower concentration compared with RVA122 and RAB1, which in turns increase the level of ADCC activation at higher concentrations. It is possible that this observation relies on the fact that RVC58 has a stronger ability in binding the specific antigen (see **Supplementary Table S6** for the IC50 of the different mAbs), thus acting at a lower concentration. Moreover, it could be that the position of the RVC58 binding to the antigenic site is not favorable for a proper and stable engagement of the Fcγ receptors and therefore for activating ADCC effectively. Taken together, the results from this and previous studies indicated that antibody-mediated effector functions may contribute to the *in vivo* efficacy of mAbs in both prophylactic and therapeutic settings. Indeed, the activation of the effector functions of the RVC20-RVC58 cocktail had already been indirectly demonstrated by comparing the therapeutic efficacy and the inflammatory pattern determined by full antibody cocktail with their LALA-mutated forms, engineered to abrogate binding to Fcγ receptors (16). Similarly, the efficacy of a mAb-based immunotherapy in controlling established lyssavirus infection in mice was reduced when using the F11-N297G mutant, displaying a defective FcRγ binding (73). The therapeutic efficacy of mAbs may therefore rely on the activation of the effector functions of infiltrating leucocytes and glial cells, possibly recruited by inflammatory mediators released by RABV-infected neurons (16). It is also worth noting that complement activation can be engaged at an early phase of the infection, as most complement proteins are synthesized by microglia and astrocytes even in the presence of the intact blood-brain barrier (BBB) (74). Moreover, infected neurons release IFN-γ, ultimately enhancing the BBB permeability (22, 75) and inducing glial differentiation toward a macrophage M1 phenotype (76–78). The immunological mechanisms involved in mAb-mediated clearance of RABV from the central nervous system are yet to be fully understood. Nevertheless, the full characterization of the effector functions mediated by mAbs included in the existing biologicals marketed for PEP might help to elucidate their potency *in vivo*. Indeed, while HRIGs are expected to activate effector functions, others such as Fab’2 products are not and are used at a higher dose in order to prevent rabies infection (1). The recent licensure of monoclonal based preparations underlines the need for a deeper characterization of their biological functions, including their interaction with the human immune system. Of note, the use of a single mAb might hamper the risk for a sub-optimal activation of effector functions, as exemplified by the poor ADCP induction of 17C7-RAB1, marketed as Rabishield (4, 5, 40, 41). In addition, some other products in the market, as the two murine mAbs M777-16-3 and 62-71-3 currently licenced as Twinrab (6), may exhibit reduced Fc-mediated functions. This could be attributed to the heterologous interaction between the murine Fc and human Fcγ receptors.

The current study highlights the significance of neutralizing and clearance activities, along with the Fc-mediated effector functions, in the mechanism of action of RVC20-RVC58. This presents a complex picture of their functions, which advocates for their combined use in the treatment of rabies. The methodology adopted in the present study might be potentially applied to any other mAb available to prevent the insurgency of RABV. However, to the best of our knowledge, no anti-rabies mAbs have shown a clearance capability *in vivo* comparable with that of the RVC20-RVC58 cocktail (3) and the mechanism behind such observation deserves further investigations.

## Data availability statement

The original contributions presented in the study are included in the article/**Supplementary Material**. Further inquiries can be directed to the corresponding author.

## Ethics statement

Procedures on Syrian hamster were authorized by the Italian Ministry of Health (Decrees 128/2011-B and 115/2014-PR).

## Author contributions

Conception and design of the study: MZ, MC, and PDB. Methodology, formal analysis and validation: MZ, MC, MG, GDM, BZ, AC, and LC. Writing original draft: MZ, MC, MG, LC, AA, and PDB. Review and editing: all. Project administration and funding acquisition: HB, DC, and PDB. All authors contributed to the article and approved the submitted version.

## References

[1] World Health Organization . Rabies vaccines: WHO position paper, April 2018 – Recommendations. Vaccine (2018) 36:5500–3. doi: 10.1016/j.vaccine.2018.06.061 30107991

[2] Hampson K Coudeville L Lembo T Sambo M Kieffer A Attlan M . Estimating the global burden of endemic canine rabies. PloS Negl Trop Dis (2015) 9:1–20. doi: 10.1371/journal.pntd.0003709 PMC4400070 25881058

[3] de Melo GD Hellert J Gupta R Corti D Bourhy H . Monoclonal antibodies against rabies: current uses in prophylaxis and in therapy. Curr Opin Virol (2022) 53:1–9. doi: 10.1016/j.coviro.2022.101204 35151116

[4] Sparrow E Torvaldsen S Newall AT Wood JG Sheikh M Kieny MP . Recent advances in the development of monoclonal antibodies for rabies post exposure prophylaxis: A review of the current status of the clinical development pipeline. Vaccine (2019) 37:A132–9. doi: 10.1016/j.vaccine.2018.11.004 30503659

[5] Rabishield. Serum Institute of India PVT. LTD (2021). Available at: https://www.seruminstitute.com/product_ind_rabishield.php.

[6] Twinrab. Zydus Corporate Park (Vaxxicare Div (2021). Available at: https://twinrab.com/.

[7] Li L Li Y Bai Y Li G Zhang J Yang L . Neutralizing antibody activity, safety and immunogenicity of human anti-rabies virus monoclonal antibody (Ormutivimab) in Chinese healthy adults: A phase IIb randomized, double-blind, parallel-controlled study. Vaccine (2022) 40:6153–62. doi: 10.1016/j.vaccine.2022.09.022 36123259

[8] zhai L Wang H Zhao W Zhang SF Miao FM Cao Y . Efficacy of ormutivimab, a novel recombinant human anti-rabies monoclonal antibody, in post-exposure prophylaxis animal models. Travel Med Infect Dis (2022) 46:102267. doi: 10.1016/j.tmaid.2022.102267 35091118

[9] Bakker ABH Python C Kissling CJ Pandya P Marissen WE Brink MF . First administration to humans of a monoclonal antibody cocktail against rabies virus: Safety, tolerability, and neutralizing activity. Vaccine (2008) 26:5922–7. doi: 10.1016/j.vaccine.2008.08.050 18804136

[10] Chao TY Ren S Shen E Moore S Zhang SF Chen L . SYN023, a novel humanized monoclonal antibody cocktail, for post-exposure prophylaxis of rabies. PloS Negl Trop Dis (2017) 11:1–20. doi: 10.1371/journal.pntd.0006133 PMC5754141 29261658

[11] Kim PK Ahn JS Kim CM Seo JM Keum SJ Lee HJ . A broad-spectrum and highly potent human monoclonal antibody cocktail for rabies prophylaxis. PloS One (2021) 16:1–21. doi: 10.1371/journal.pone.0256779 PMC8409651 34469480

[12] Sun L Liu Y Li C Li D Liang M . Development of recombinant human monoclonal antibody cocktail for post-exposure rabies prophylaxis. Chin J Virol (2016) 32:399–403. doi: 10.3390/v15010088 29979531

[13] Dietzschold B Kao M Zheng YM Chen ZY Maul G Fu ZF . Delineation of putative mechanisms involved in antibody-mediated clearance of rabies virus from the central nervous system. Proc Natl Acad Sci U.S.A. (1992) 89:7252–6. doi: 10.1073/pnas.89.15.7252 PMC49684 1496020

[14] Du Pont V Plemper RK Schnell MJ . Status of antiviral therapeutics against rabies virus and related emerging lyssaviruses. Curr Opin Virol (2019) 35:1–13. doi: 10.1016/j.coviro.2018.12.009 30753961 PMC6556400

[15] Willoughby RE Tieves KS Hoffman GM Ghanayem NS Amlie-Lefond CM Schwabe MJ . Survival after treatment of rabies with induction of coma. N Engl J Med (2005) 352:2508–14. doi: 10.1056/nejmoa050382 15958806

[16] Dias de Melo G Sonthonnax F Lepousez G Jouvion G Minola A Zatta F . A combination of two human monoclonal antibodies cures symptomatic rabies. EMBO Mol Med (2020) 12:1–10. doi: 10.15252/emmm.202012628 PMC7645379 32945125

[17] De Benedictis P Minola A Rota Nodari E Aiello R Zecchin B Salomoni A . Development of broad-spectrum human monoclonal antibodies for rabies post-exposure prophylaxis. EMBO Mol Med (2016) 8:407–21. doi: 10.15252/emmm.201505986 PMC4818751 26992832

[18] Hellert J Buchrieser J Larrous F Minola A de Melo GD Soriaga L . Structure of the prefusion-locking broadly neutralizing antibody RVC20 bound to the rabies virus glycoprotein. Nat Commun (2020) 11:25–8. doi: 10.1038/s41467-020-14398-7 PMC6992781 32001700

[19] Ng WM Fedosyuk S English S Augusto G Berg A Thorley L . Structure of trimeric pre-fusion rabies virus glycoprotein in complex with two protective antibodies. Cell Host Microbe (2022) 30:1219–1230.e7. doi: 10.1016/j.chom.2022.07.014 35985336 PMC9605875

[20] Callaway HM Zyla D Larrous F de Melo GD Hastie KM Avalos RD . Structure of the rabies virus glycoprotein trimer bound to a prefusion-specific neutralizing antibody. Sci Adv (2022) 8:1–13. doi: 10.1126/sciadv.abp9151 PMC9205594 35714192

[21] Yang F Lin S Ye F Yang J Qi J Chen Z . Structural analysis of rabies virus glycoprotein reveals pH-dependent conformational changes and interactions with a neutralizing antibody. Cell Host Microbe (2020) 27:441–453.e7. doi: 10.1016/j.chom.2019.12.012 32004500

[22] Katz ISS Guedes F Fernandes ER dos Ramos Silva S . Immunological aspects of rabies: a literature review. Arch Virol (2017) 162:3251–68. doi: 10.1007/s00705-017-3484-0 28726129

[23] Feige L Sáenz-de-Santa-María I Regnault B Lavenir R Lepelletier A Halacu A . Transcriptome profile during rabies virus infection: identification of human CXCL16 as a potential new viral target. Front Cell Infect Microbiol (2021) 11:761074. doi: 10.3389/fcimb.2021.761074 34804996 PMC8602097

[24] Besson B Kim S Kim T Ko Y Lee S Larrous F . Kinome-wide RNA interference screening identifies mitogen-activated protein kinases and phosphatidylinositol metabolism as key factors for rabies virus infection. mSphere (2019) 4:e00047-19. doi: 10.1128/MSPHERE.00047-19/SUPPL_FILE/MSPHERE.00047-19-SF006.PDF 31118297 PMC6531879

[25] Stettler K Beltramello M Espinosa DA Graham V Cassotta A Bianchi S . Specificity, cross-reactivity, and function of antibodies elicited by Zika virus infection. Science (2016) 353:823–6. doi: 10.1126/science.aaf8505 27417494

[26] Arnold K Bordoli L Kopp J Schwede T . The SWISS-MODEL workspace: A web-based environment for protein structure homology modelling. Bioinformatics (2006) 22:195–201. doi: 10.1093/bioinformatics/bti770 16301204

[27] Biasini M Bienert S Waterhouse A Arnold K Studer G Schmidt T . SWISS-MODEL: Modelling protein tertiary and quaternary structure using evolutionary information. Nucleic Acids Res (2014) 42:252–8. doi: 10.1093/nar/gku340 PMC4086089 24782522

[28] Sivasubramanian A Sircar A Chaudhury S Gray JJ . Toward high-resolution homology modeling of antibody F v regions and application to antibody-antigen docking. Proteins Struct Funct Bioinforma (2009) 74:497–514. doi: 10.1002/prot.22309 PMC2909601 19062174

[29] Van Zundert GCP Rodrigues JPGLM Trellet M Schmitz C Kastritis PL Karaca E . The HADDOCK2.2 web server: user-friendly integrative modeling of biomolecular complexes. J Mol Biol (2016) 428:720–5. doi: 10.1016/j.jmb.2015.09.014 26410586

[30] Honorato RV Koukos PI Jiménez-García B Tsaregorodtsev A Verlato M Giachetti A . Structural biology in the clouds: the weNMR-EOSC ecosystem. Front Mol Biosci (2021) 8:729513. doi: 10.3389/fmolb.2021.729513 34395534 PMC8356364

[31] Patey GN Valleau JP . The free energy of spheres with dipoles: Monte Carlo with multistage sampling. Chem Phys Lett (1973) 21:297–300. doi: 10.1016/0009-2614(73)80139-3

[32] Torrie GM Valleau JP . Monte Carlo free energy estimates using non-Boltzmann sampling: Application to the sub-critical Lennard-Jones fluid. Chem Phys Lett (1974) 28:578–81. doi: 10.1016/0009-2614(74)80109-0

[33] Torrie GM Valleau JP . Nonphysical sampling distributions in Monte Carlo free-energy estimation: Umbrella sampling. J Comput Phys (1977) 23:187–99. doi: 10.1016/0021-9991(77)90121-8

[34] Mills M Andricioaei I . An experimentally guided umbrella sampling protocol for biomolecules. J Chem Phys (2008) 129:114101. doi: 10.1063/1.2976440 19044944 PMC2736582

[35] Lemkul JA Bevan DR . Assessing the stability of Alzheimer’s amyloid protofibrils using molecular dynamics. J Phys Chem B (2010) 114:1652–60. doi: 10.1021/JP9110794/SUPPL_FILE/JP9110794_SI_001.PDF 20055378

[36] Greer AM Wu N Putnam AL Woodruff PG Wolters P Kinet JP . Serum IgE clearance is facilitated by human FcϵRI internalization. J Clin Invest (2014) 124:1187–98. doi: 10.1172/JCI68964 PMC3938266 24569373

[37] Thompson BS Moesker B Smit JM Wilschut J Diamond MS Fremont DH . A therapeutic antibody against West Nile virus neutralizes infection by blocking fusion within endosomes. PloS Pathog (2009) 5:e1000453. doi: 10.1371/journal.ppat.1000453 19478866 PMC2679195

[38] Galeotti C Karnam A Das M Kaveri SV Bayry J . Acid stripping of surface IgE antibodies bound to FcϵRI is unsuitable for the functional assays that require long-term culture of basophils and entire removal of surface IgE. Int J Mol Sci (2020) 21. doi: 10.3390/ijms21020510 PMC7014331 31941161

[39] Lu X Xiao H Li S Pang X Song J Liu S . Double lock of a human neutralizing and protective monoclonal antibody targeting the yellow fever virus envelope. Cell Rep (2019) 26:438–446.e5. doi: 10.1016/j.celrep.2018.12.065 30625326

[40] Sloan SE Hanlon C Weldon W Niezgoda M Blanton J Self J . Identification and characterization of a human monoclonal antibody that potently neutralizes a broad panel of rabies virus isolates. Vaccine (2007) 25:2800–10. doi: 10.1016/j.vaccine.2006.12.031 17240489

[41] Gogtay NJ Munshi R Narayana DHA Mahendra BJ Kshirsagar V Gunale B . Comparison of a novel human rabies monoclonal antibody to human rabies immunoglobulin for postexposure prophylaxis: A phase 2/3, randomized, single-blind, noninferiority, controlled study. Clin Infect Dis (2018) 66:387–95. doi: 10.1093/cid/cix791 29020321

[42] Forthal DN . Functions of antibodies. Antibodies Infect Dis (2015), 45–8. doi: 10.1128/9781555817411.ch2

[43] McCullough KC Smale CJ Carpenter WC Crowther JR Brocchi E De Simone F . Conformational alteration in foot-and-mouth disease virus virion capsid structure after complexing with monospecific antibody. Immunology (1987) 60:75–82. 3028937 PMC1453358

[44] Hernandez R Paredes A Brown DT . Sindbis virus conformational changes induced by a neutralizing anti-E1 monoclonal antibody. J Virol (2008) 82:5750–60. doi: 10.1128/jvi.02673-07 PMC2395122 18417595

[45] Liu X Lin H Tang Q Li C Yang S Wang Z . Characterization of a human antibody fragment fab and its calcium phosphate nanoparticles that inhibit rabies virus infection with vaccine. PloS One (2011) 6:e19848. doi: 10.1371/journal.pone.0019848 21573024 PMC3090417

[46] Ku Z Ye X Shi J Wang X Liu Q Huang Z . Single Neutralizing Monoclonal Antibodies Targeting the VP1 GH Loop of Enterovirus 71 Inhibit both Virus Attachment and Internalization during Viral Entry. J Virol (2015) 89:12084–95. doi: 10.1128/jvi.02189-15 PMC4645313 26401034

[47] Booy FP Roden RBS Greenstone HL Schiller JT Trus BL . Two antibodies that neutralize papillomavirus by different mechanisms show distinct binding patterns at 13 Å resolution. J Mol Biol (1998) 281:95–106. doi: 10.1006/jmbi.1998.1920 9680478

[48] Liu X Chen J Wang Z Wang D He M Qian C . Neutralization sites of human papillomavirus-6 relate to virus attachment and entry phase in viral infection. Emerg Microbes Infect (2019) 8:1721–33. doi: 10.1080/22221751.2019.1694396 PMC6883418 31769733

[49] Edwards MJ Dimmock NJ . Hemagglutinin 1-specific immunoglobulin G and fab molecules mediate postattachment neutralization of influenza A virus by inhibition of an early fusion event. J Virol (2001) 75:10208–18. doi: 10.1128/jvi.75.21.10208-10218.2001 PMC114595 11581389

[50] Barbey-Martin C Gigant B Bizebard T Calder LJ Wharton SA Skehel JJ . An antibody that prevents the hemagglutinin low pH fusogenic transition. Virology (2002) 294:70–4. doi: 10.1006/viro.2001.1320 11886266

[51] Kaufmann B Nybakken GE Chipman PR Zhang W Diamond MS Fremont DH . West Nile virus in complex with the Fab fragment of a neutralizing monoclonal antibody. Proc Natl Acad Sci U.S.A. (2006) 103:12400–4. doi: 10.1073/pnas.0603488103 PMC1567891 16895988

[52] Ekiert DC Bhabha G Elsliger M Friesen RHE Jongeneelen M Throsby M . Antibody recognition of a highly conserved influenza virus epitope : implications for universal prevention and therapy. Science (2009) 324:246–51. doi: 10.1126/science.1171491.Antibody PMC2758658 19251591

[53] Kallewaard NL Corti D Collins PJ Neu U McAuliffe JM Benjamin E . Structure and function analysis of an antibody recognizing all influenza A subtypes. Cell (2016) 166:596–608. doi: 10.1016/j.cell.2016.05.073 27453466 PMC4967455

[54] Dietzschold B . Antibody-mediated clearance of viruses from the mamMalian central nervous system. Trends Microbiol (1993) 1:63–6. doi: 10.1016/0966-842X(93)90035-P PMC7133251 8044464

[55] Hughey PG Roberts PC Holsinger LJ Zebedee SL Lamb RA Compans RW . Effects of antibody to the influenza A virus M2 protein on M2 surface expression and virus assembly. Virology (1995) 212:411–21. doi: 10.1006/viro.1995.1498 7571410

[56] Kajihara M Marzi A Nakayama E Noda T Kuroda M Manzoor R . Inhibition of marburg virus budding by nonneutralizing antibodies to the envelope glycoprotein. J Virol (2012) 86:13467–74. doi: 10.1128/jvi.01896-12 PMC3503067 23035224

[57] Jin J Galaz-Montoya JG Sherman MB Sun SY Goldsmith CS O’Toole ET . Neutralizing antibodies inhibit chikungunya virus budding at the plasma membrane. Cell Host Microbe (2018) 24:417–428.e5. doi: 10.1016/j.chom.2018.07.018 30146390 PMC6137268

[58] Corboba P Grutadauria S Cuffini C Zapata M . Neutralizing monoclonal antibody to the E1 glycoprotein epitope of rubella virus mediates virus arrest in VERO cells. Viral Immunol (2000) 13:83–92. doi: 10.1089/vim.2000.13.83 10733171

[59] Tuffereau C Bénéjean J Blondel D Kieffer B Flamand A . Low-affinity nerve-growth factor receptor (P75NTR) can serve as a receptor for rabies virus. EMBO J (1998) 17:7250–9. doi: 10.1093/emboj/17.24.7250 PMC1171071 9857182

[60] Coulon P Ternaux J-P Flamand A Tuffereau C . An avirulent mutant of rabies virus is unable to infect motoneurons *in vivo* and *in vitro* . J Virol (1998) 72:273–8. doi: 10.1128/jvi.72.1.273-278.1998 PMC109373 9420224

[61] Faber M Li J Kean RB Hooper DC Alugupalli KR Dietzschold B . Effective preexposure and postexposure prophylaxis of rabies with a highly attenuated recombinant rabies virus. Proc Natl Acad Sci U.S.A. (2009) 106:11300–5. doi: 10.1073/pnas.0905640106 PMC2706273 19581599

[62] Yin J Wang X Mao R Zhang Z Gao X Luo Y . Research advances on the interactions between rabies virus structural proteins and host target cells: Accrued knowledge from the application of reverse genetics systems. Viruses (2021) 13. doi: 10.3390/v13112288 PMC8617671 34835093

[63] Seif I Coulon P Rollin PE Flamand A . Rabies virulence: effect on pathogenicity and sequence characterization of rabies virus mutations affecting antigenic site III of the glycoprotein. J Virol (1985) 53:926–34. doi: 10.1128/jvi.53.3.926-934.1985 PMC254728 2579247

[64] Tao L Ge J Wang X Zhai H Hua T Zhao B . Molecular basis of neurovirulence of flury rabies virus vaccine strains: importance of the polymerase and the glycoprotein R333Q mutation. J Virol (2010) 84:8926–36. doi: 10.1128/jvi.00787-10 PMC2919041 20538851

[65] Shuai L Feng N Wang X Ge J Wen Z Chen W . Genetically modified rabies virus ERA strain is safe and induces long-lasting protective immune response in dogs after oral vaccination. Antiviral Res (2015) 121:9–15. doi: 10.1016/j.antiviral.2015.06.011 26093157

[66] Jayakar HR Jeetendra E Whitt MA . Rhabdovirus assembly and budding. Virus Res (2004) 106:117–32. doi: 10.1016/j.virusres.2004.08.009 15567492

[67] Cadd TL Skoging U Liljestrom P . Budding of enveloped viruses from the plasma membrane. Bioessays. (1997) 19(11):993–1000. doi: 10.1002/bies.950191109 PMC7161837 9394621

[68] Mebatsion T Konig M Conzelmann KK . Budding of rabies virus particles in the absence of the spike glycoprotein. Cell (1996) 84:941–51. doi: 10.1016/S0092-8674(00)81072-7 8601317

[69] Pinto D Sauer MM Czudnochowski N Low JS Alejandra Tortorici M Housley MP . Broad betacoronavirus neutralization by a stem helix–specific human antibody. Science (2021) 373:1109–16. doi: 10.1126/science.abj3321 PMC9268357 34344823

[70] Corti D Cameroni E Guarino B Kallewaard NL Zhu Q Lanzavecchia A . Tackling influenza with broadly neutralizing antibodies. Curr Opin Virol (2017) 24:60–9. doi: 10.1016/j.coviro.2017.03.002 PMC7102826 28527859

[71] Corti D Purcell LA Snell G Veesler D . Tackling COVID-19 with neutralizing monoclonal antibodies. Cell (2021) 184:3086–108. doi: 10.1016/j.cell.2021.05.005 PMC8152891 34087172

[72] Corti D Lanzavecchia A . Broadly neutralizing antiviral antibodies. Annu Rev Immunol (2013) 31:705–42. doi: 10.1146/annurev-immunol-032712-095916 23330954

[73] Huaman C Mastraccio KE Coggins SA Hussain I Yan L Ahmed AE . Control of established, CNS-resident lyssavirus infection by an adaptive immune response stimulated by single-dose monoclonal antibody therapy. J Immunol (2022) 208:64. doi: 10.4049/jimmunol.208.Supp.64.20

[74] Dalakas MC Alexopoulos H Spaeth PJ . Complement in neurological disorders and emerging complement-targeted therapeutics. Nat Rev Neurol (2020) 16:601–17. doi: 10.1038/s41582-020-0400-0 PMC7528717 33005040

[75] Feige L Zaeck LM Sehl-Ewert J Finke S Bourhy H . Innate immune signaling and role of glial cells in herpes simplex virus-and rabies virus-induced encephalitis. Viruses (2021) 13. doi: 10.3390/v13122364 PMC8708193 34960633

[76] Chhatbar C Prinz M . The roles of microglia in viral encephalitis: from sensome to therapeutic targeting. Cell Mol Immunol (2021) 18:250–8. doi: 10.1038/s41423-020-00620-5 PMC7802409 33437050

[77] Quan Y Möller T Weinstein JR . Regulation of Fcγ receptors and immunoglobulin G-mediated phagocytosis in mouse microglia. Neurosci Lett (2009) 464:29–33. doi: 10.1016/j.neulet.2009.08.013 19679164 PMC2747046

[78] Chauhan P Hu S Sheng WS Prasad S Lokensgard JR . Modulation of microglial cell fcγ Receptor expression following viral brain infection. Sci Rep (2017) 7:1–11. doi: 10.1038/srep41889 28165503 PMC5292951

