# Supplementary figures and images for: Antiviral mechanisms of two broad-spectrum monoclonal antibodies for rabies prophylaxis and therapy
Source: Front Immunol. 2023 Aug 10;14:1186063. doi: 10.3389/fimmu.2023.1186063 (PMC10449259; doi:10.3389/fimmu.2023.1186063)

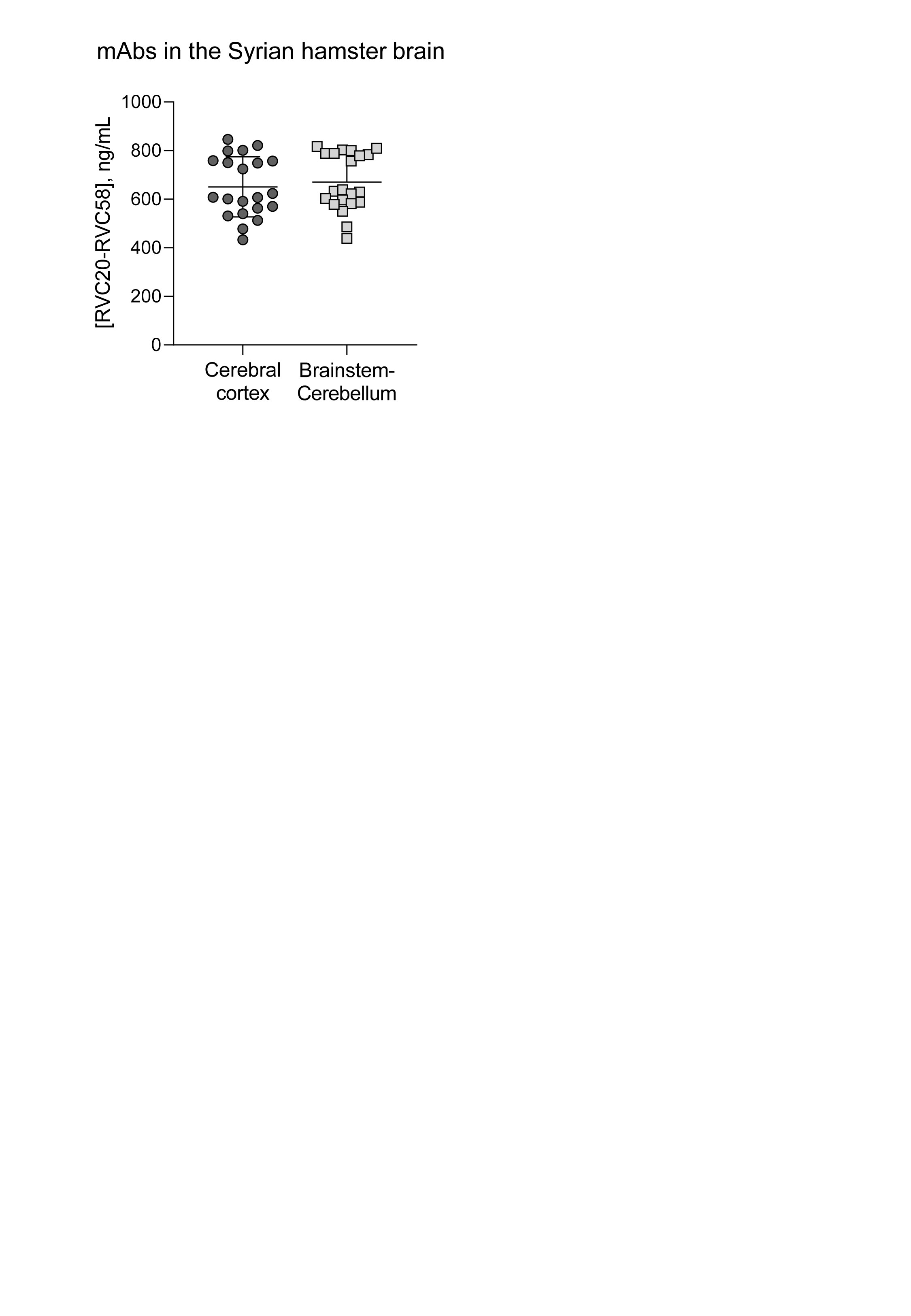

Supplement: Supplementary file 1 [file Image_1.jpeg]

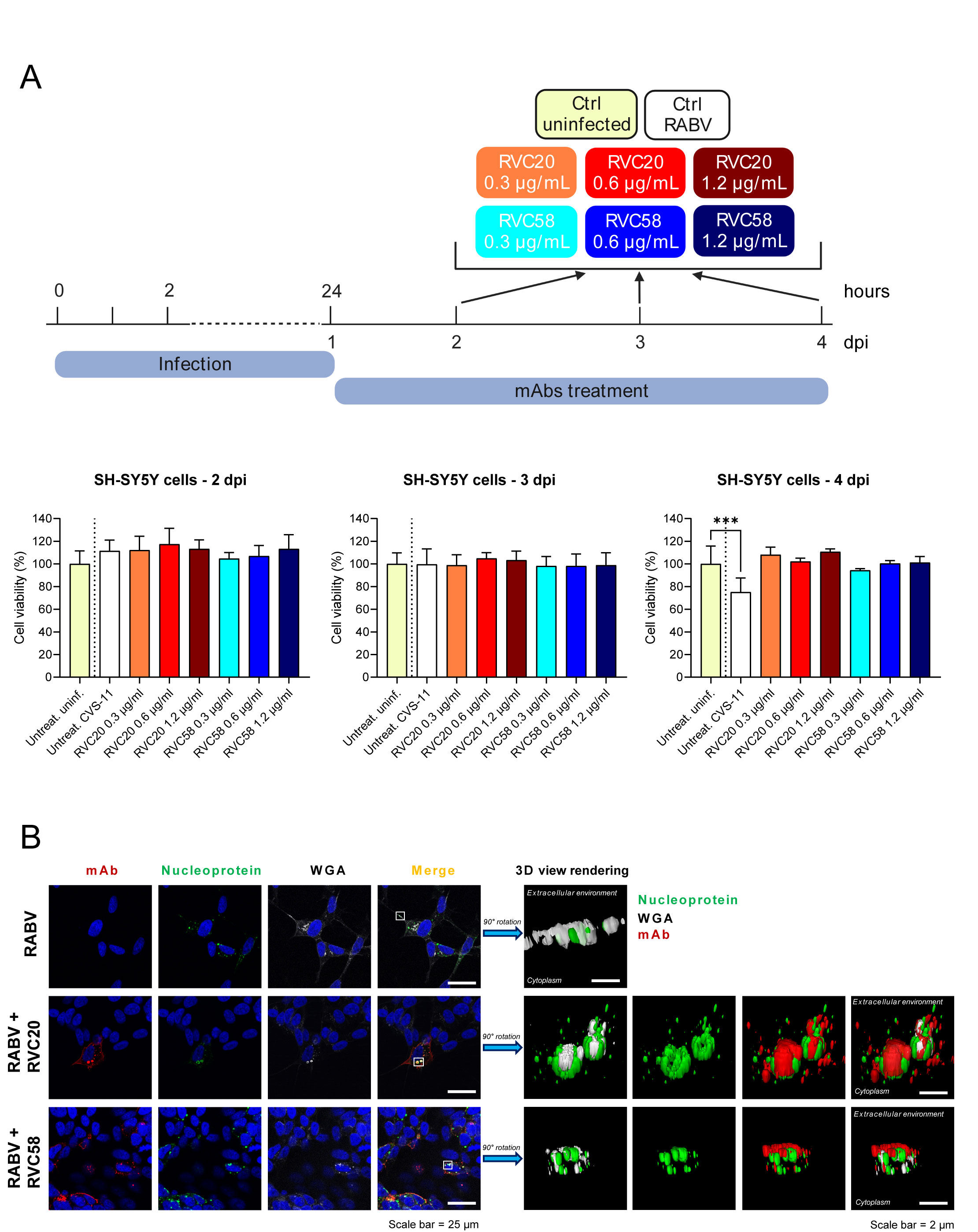

Supplement: Supplementary file 2 [file Image_2.jpeg]

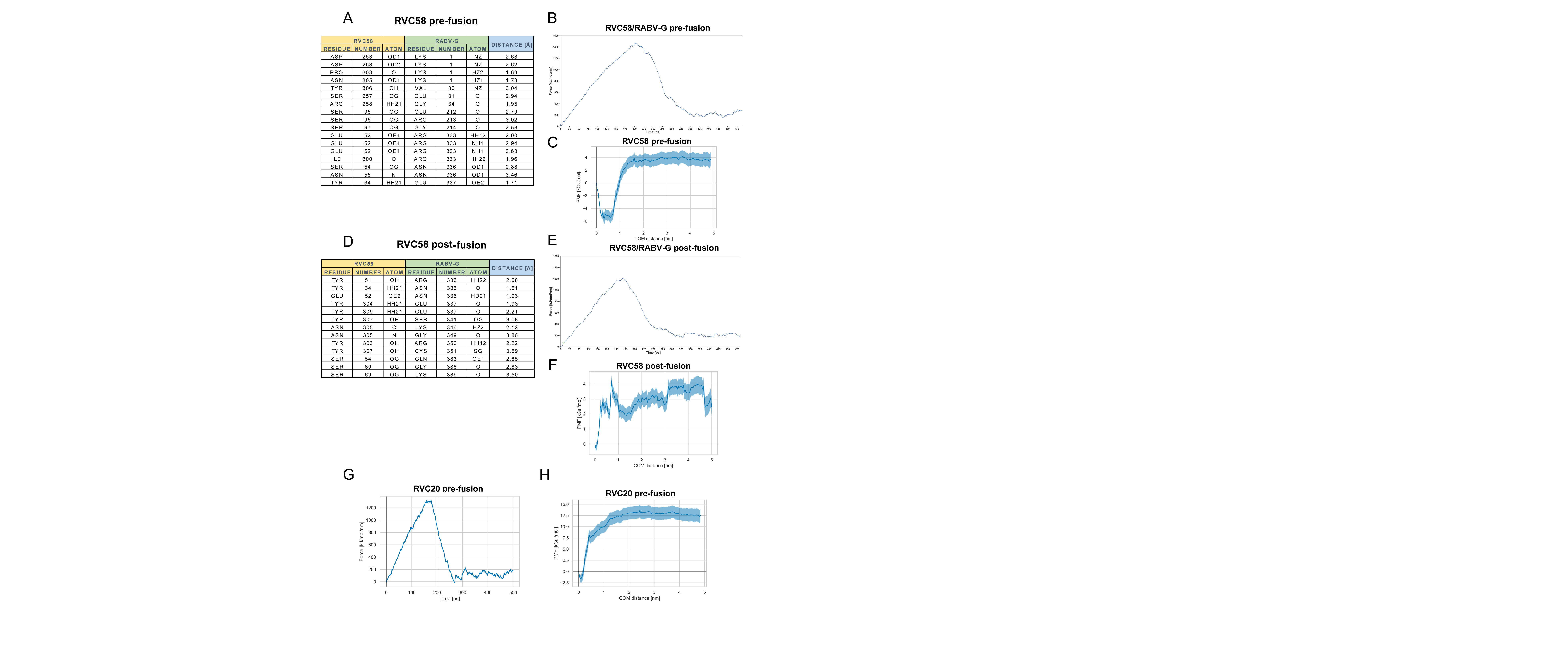

Supplement: Supplementary file 3 [file Image_3.jpeg]
