# Supplementary material for: Antiviral mechanisms of two broad-spectrum monoclonal antibodies for rabies prophylaxis and therapy
Source: Front Immunol. 2023 Aug 10;14:1186063. doi: 10.3389/fimmu.2023.1186063 (PMC10449259; doi:10.3389/fimmu.2023.1186063)
Supplement: Supplementary file 5 [file DataSheet_1.docx]

Supplementary Material

Antiviral mechanisms of two broad-spectrum monoclonal antibodies for rabies prophylaxis and therapy

Maira Zorzan^1†^, Martina Castellan^1†^, Matteo Gasparotto^2^, Guilherme Dias De Melo^3^, Barbara Zecchin^1^, Stefania Leopardi^1^, Alex Chen^4^, Antonio Rosato^5,6^, Alessandro Angelini^7,8^, Hervé Bourhy^3^, Davide Corti^9^, Laura Cendron^2^ & Paola De Benedictis^1^*****

^1^Laboratory for Emerging Viral Zoonoses, FAO and National Reference Centre for Rabies, Department for Research and Innovation, Istituto Zooprofilattico Sperimentale delle Venezie, Legnaro, Italy

^2^Department of Biology, University of Padua, Via U. Bassi 58, 35131 Padova, Italy

^3^Institut Pasteur, Université Paris Cité, Lyssavirus Epidemiology and Neuropathology Unit, WHO Collaborating Centre for Reference and Research on Rabies, Paris, F-75015, France

^4^Vir Biotechnology, San Francisco, CA, USA

^5^Department of Surgery, Oncology and Gastroenterology, University of Padua, Padua, Italy

^6^Immunology and Molecular Oncology Diagnostics, Veneto Institute of Oncology, Padua, Italy

^7^Department of Molecular Sciences and Nanosystems, Ca’ Foscari University of Venice, Via Torino 155, 30172 Mestre, Italy

^8^European Centre for Living Technology (ECLT), Ca’ Bottacin, Dorsoduro 3911, Calle Crosera, 30123 Venice, Italy

^9^Humabs BioMed SA, a subsidiary of Vir Biotechnology, Bellinzona, Switzerland.

† These authors equally contributed to the study

*** Correspondence:**Paola De Benedictis
pdebenedictis@izsvenezie.it

# Supplementary Figures, Tables and Videos

## Supplementary Figures

**Supplementary Figure S1. Human IgG quantification in the Syrian hamster’s cerebral cortex and brainstem/cerebellum.** 21 brains collected at different time post–treatment with the RVC20-RVC58 cocktail have been analyzed through ELISA and quantified (ng/mL). Mean ± SD is represented. Average concentration in the cerebral cortex = 650.791 ng/mL; average concentration in the brainstem/cerebellum = 670.229 ng/mL.

**Supplementary Figure S2.** **(A)** **SH-SY5Y viability after RABV infection and treatment.** Cell viability percentage of SH-SY5Y RABV (CVS-11)-infected cells (MOI 0.1), treated 24 hours post-infection with RVC20 or RVC58 at different concentrations. Bars represent mean ± SD. Unpaired *t*-tests of Untreated Uninfected cells *Vs* Untreated CVS-11-infected cells ***p<0.001). Cartoon created with BioRender.com (Agreement number EH251OWMK2). **(B)** **3D view rendering of rabies nucleoprotein and WGA in membrane portions of RABV-infected SH-SY5Y cells treated with RVC20 or RVC58**. As for previous experiments, cells were treated with RVC20 (0.6 µg/mL) 1 dpi with RABV (CVS-11, MOI 0.1). Cells where then fixed after 24 hours of treatment (2 dpi). Cells were stained with FITC-conjugated anti-nucleoprotein antibody and Alexa Fluor 568-conjugated anti-Human IgG antibody. WGA was used as cell membrane marker. Z-stack images were acquired by confocal microscope, and then processed for 3D visualization through ImageJ software. The area processed for the 3D rendering is highlighted with a white box. Green: viral nucleoprotein; white: WGA membrane marker. Scale bar = 25 µm. See Supplementary Video 1-6 for the rotating view of the 3D reconstructions of RABV (1-2), RABV+RVC20 (3-4) and RABV+RVC58 (5-6).

**Supplementary Figure S3.** **Results of the** **umbrella sampling of RABV-G bound to characterized scFv.** Residues engaged in the interaction interface between RVC58 and RABV-G in the pre-fusion (Table **A**) and post-fusion (Table **D**) conformations are listed in the tables. Plots of pulling force versus time of the dissociation simulation of RVC58 from RABV-G and predicted PMF versus the distance between center of masses (COM) in the pre-fusion **(B** and **C)** and post-fusion **(E** and **F)** complexes are shown. Corresponding calculations made for RVC20 bound to RABV-G are shown in panels **G** and **H**. Intervals in graphs **C**, **F** and **H** represent calculated SD.

## Supplementary Tables

**Supplementary Table S1.** RVC20-RVC58 cocktail mediates RABV (Tha-GFP) neutralization and infection treatment in SK-N-SH cells infected with a multiplicity of infection (MOI) of 0.1 or 1 at 4 and 6 dpi. Raw data related to Figure 1A-B. In order to calculate the increase rate described in the result section 3.1, the percentage of infected cells after mAbs treatment has been divided by the percentage of infected cells at 1dpi (see ‘treat 0.1 - blank’ and ‘treat 1 - blank’).

**Supplementary Table S2.** RVC20-RVC58 cocktail mediates RABV (Tha-GFP) infection treatment in SK-N-SH cells infected with a multiplicity of infection (MOI) of 0.1 at 4 days post-infection (dpi). Related to Figure 1B.

| RVC20+RVC58  (µg/mL) | MOI 0.1 (4 dpi) | | |
| --- | --- | --- | --- |
|  | total cells/well (x10^3^) | infected cells/well (x10^3^) | infected cells/well (%) |
| 100+100 (4 dpi) | 17.1 (±1.1) | 1.1 (±0.3) | 6.3 (±2.1) |
| 10+10 (4 dpi) | 16.3 (±1.5) | 0.9 (±0.1) | 6.1 (±1.0) |
| 1+1 (4 dpi) | 15.9 (±0.6) | 1.0 (±0.1) | 6.5 (±0.5) |
| 0.1+0.1 | 14.5 (±0.9) | 1.0 (±0.2) | 7.0 (±0.8) |
| infected, non-treated (1 dpi) | 3.6 (±0.5) | 0.09 (±0.01) | 2.6 (±0.3) |
| infected, non-treated (4 dpi) | 8.0 (±0.6) | 7.3 (±0.7) | 95.8 (±7.4) |
| non-infected, non-treated (4 dpi) | 9.5 (±1.0) | - | - |

Values are expressed as mean (± SD)

**Supplementary Table S3.** RVC20-RVC58 cocktail mediates RABV (Tha-GFP) infection treatment in SK-N-SH cells infected with a multiplicity of infection (MOI) of 1 at 4 dpi. Related to Figure 1B.

| RVC20+RVC58  (µg/mL) | MOI 1 (4 dpi) | | |
| --- | --- | --- | --- |
|  | total cells/well (x10^3^) | infected cells/well (x10^3^) | infected cells/well (%) |
| 100+100 (4 dpi) | 8.9 (±0.3) | 3.5 (±0.2) | 39.4 (±2.3) |
| 10+10 (4 dpi) | 10.9 (±0.7) | 4.6 (±0.2) | 41.8 (±1.6) |
| 1+1 (4 dpi) | 11.2 (±0.6) | 4.5 (±0.5) | 39.7 (±2.1) |
| 0.1+0.1 (4 dpi) | 12.0 (±0.7) | 4.4 (±0.2) | 36.9 (±3.2) |
| infected, non-treated (1 dpi) | 3.1 (±0.4) | 0.7 (±0.04) | 21.9 (±2.5) |
| infected, non-treated (4 dpi) | 5.1 (±0.7) | 5.0 (±0.6) | 102.2 (±9.5) |
| non-infected, non-treated (4 dpi) | 9.5 (± 1.0) | - | - |

Values are expressed as mean (± SD)

**Supplementary Table S4.** RVC20-RVC58 cocktail mediates RABV (Tha-GFP) infection treatment in SK-N-SH cells infected with a multiplicity of infection (MOI) of 0.1 at 6 dpi. Related to Figure 1B.

| RVC20+RVC58  (µg/mL) | MOI 0.1 (6 dpi) | | |
| --- | --- | --- | --- |
|  | total cells/well (x10^3^) | infected cells/well (x10^3^) | infected cells/well (%) |
| 100+100 (6 dpi) | 43.2 (±1.5) | 1.9 (±0.3) | 4.4 (±0.5) |
| 10+10 (6 dpi) | 41.4 (±1.2) | 1.6 (±0.2) | 4.0 (±0.3) |
| 1+1 (6dpi) | 39.3 (±2.9) | 1.6 (±0.3) | 4.1 (±0.6) |
| 0.1+0.1 (6 dpi) | 26.7 (±1.5) | 1.1 (±0.2) | 4.1 (±0.7) |
| infected, non-treated (1 dpi) | 3.6 (±0.5) | 0.09 (±0.01) | 2.6 (±0.3) |
| infected, non-treated (6 dpi) | 9.4 (±2.1) | 8.9 (±1.5) | 91.3 (±7.9) |
| non-infected, non-treated (6 dpi) | 20.8 (±3.6) | - | - |

Values are expressed as mean (± SD)

**Supplementary Table S5.** RVC20-RVC58 cocktail mediates RABV (Tha-GFP) infection treatment in SK-N-SH cells infected with a multiplicity of infection (MOI) of 1 at 6 dpi. Related to Figure 1B.

| RVC20+RVC58  (µg/mL) | MOI 1 (6 dpi) | | |
| --- | --- | --- | --- |
|  | total cells/well (x10^3^) | infected cells/well (x10^3^) | infected cells/well (%) |
| 100+100 (6 dpi) | 16.1 (±2.0) | 3.9 (±0.4) | 24.3 (±2.3) |
| 10+10 (6 dpi) | 24.7 (±0.5) | 6.3 (±0.7) | 25.3 (±2.9) |
| 1+1 (6 dpi) | 27.5 (±1.8) | 7.2 (±0.4) | 26.2 (±3.1) |
| 0.1+0.1 (6 dpi) | 29.9 (±3.6) | 7.5 (±1.2) | 25.1 (±1.9) |
| infected, non-treated (1 dpi) | 3.1 (±0.4) | 0.7 (±0.04) | 21.9 (±2.5) |
| infected, non-treated (6 dpi) | 5.9 (±1.5) | 5.9 (±1.1) | 99.1 (±3.7) |
| non-infected, non-treated (6 dpi) | 20.8 (±3.6) | - | - |

Values are expressed as mean (± SD)

**Supplementary Table S6.** Neutralization ability of tested mAbs and their Fab forms expressed as IC50 (ng/mL)

| mAb | **IC50 (ng/mL)** |
| --- | --- |
| RVC20 | 47.26 |
| Fab20 | 17640.68 |
| RVC58 | 3.57 |
| Fab58 | 153.84 |
| 17C7 – RAB1 | 29 |
| RVA122 | 1.2 |

**Supplementary Table S7.** Detailed statistical results related to Figure 4D; one-way ANOVA with Tukey’s multiple comparisons. The boxes highlighted in grey indicate that the second mAb is statistically more powerful than the first one. *p<0.05; **p<0.01; ***p<0.001; ****p<0.0001. Antibody concentration: 4.44 µg/mL (log10=0.65).

|  | **CDC – % of cell viability** |  | **ADCC – fold of induction** |  | **ADCP – fold of induction** |  |
| --- | --- | --- | --- | --- | --- | --- |
| **Comparison** | P values | Stars | P values | Stars | P values | Stars |
| RVC20 vs Fab20 | <0.0001 | **** | <0.0001 | **** | <0.0001 | **** |
| RVC58 vs Fab58 | <0.0001 | **** | 0.0262 | * | 0.3821 | ns |
| RVC20 vs RVC58 | <0.0001 | **** | <0.0001 | **** | <0.0001 | **** |
| RVC20 vs RVC20+RVC58 | 0.0074 | ** | 0.4098 | ns | 0.0001 | **** |
| RVC58 vs RVC20+RVC58 | 0.8412 | ns | <0.0001 | **** | <0.0001 | **** |
| RVC20 vs 17C7 - RAB1 | <0.0001 | **** | 0.0032 | ** | <0.0001 | **** |
| RVC20 vs RVA122 | <0.0001 | **** | 0.0001 | *** | <0.0001 | **** |
| RVC58 vs 17C7 - RAB1 | 0.0002 | *** | 0.0785 | ns | 0.095 | ns |
| RVC58 vs RVA122 | <0.0001 | **** | 0.9679 | ns | 0.0732 | ns |
| RVC20+RVC58 vs 17C7 - RAB1 | <0.0001 | **** | 0.0002 | *** | <0.0001 | **** |
| RVC20+RVC58 vs RVA122 | <0.0001 | **** | <0.0001 | **** | <0.0001 | **** |

**Supplementary Table S8.** Detailed statistical results related to Figure 4E; one-way ANOVA with Tukey’s multiple comparisons. *p<0.05; **p<0.01; ***p<0.001; ****p<0.0001. Antibody concentration 0.16 µg/mL (log10=-0.78).

|  | **CDC – % of cell viability** |  | **ADCC – fold of induction** |  | **ADCP – fold of induction** |  |
| --- | --- | --- | --- | --- | --- | --- |
| **Comparison** | P values | Stars | P values | Stars | P values | Stars |
| RVC20 vs Fab20 | >0.9999 | ns | <0.0001 | **** | <0.0001 | **** |
| RVC58 vs Fab58 | 0.9995 | ns | 0.0094 | ** | 0.9876 | ns |
| RVC20 vs RVC58 | 0.9852 | ns | <0.0001 | **** | <0.0001 | **** |
| RVC20 vs RVC20+RVC58 | 0.9985 | ns | <0.0001 | **** | <0.0001 | **** |
| RVC58 vs RVC20+RVC58 | 0.8093 | ns | 0.0416 | * | 0.9998 | ns |
| RVC20 vs 17C7 - RAB1 | 0.9973 | ns | <0.0001 | **** | 0.0006 | *** |
| RVC20 vs RVA122 | 0.4515 | ns | <0.0001 | **** | <0.0001 | **** |
| RVC58 vs 17C7 - RAB1 | >0.9999 | ns | 0.1153 | ns | 0.8406 | ns |
| RVC58 vs RVA122 | 0.1029 | ns | 0.0163 | * | >0.9999 | ns |
| RVC20+RVC58 vs 17C7 - RAB1 | 0.9032 | ns | >0.9999 | ns | 0.9436 | ns |
| RVC20+RVC58 vs RVA122 | 0.8089 | ns | 0.9573 | ns | 0.9991 | ns |

**Supplementary Table S9.** Glycan profiling of RVC20 and RVC58 as determined by liquid chromatography-mass spectrometry.

| RVC20 | | | | | | |
| --- | --- | --- | --- | --- | --- | --- |
| **Glycan** | **Abundance (%)** | Theoretical mass (Da) | Observed mass (Da) | Delta (Da) | RT (min) | |
| **G0F-Gn** | **2.5** | 2429.9593 | 2429.9578 | -0.0015 | 20.7 | |
| **G0F** | **59.7** | 2633.0387 | 2633.0518 | 0.0131 | 20.7 | |
| **G1F** | **31.3** | 2795.0915 | 2795.0967 | 0.0052 | 20.7 | |
| **G2F** | **4.8** | 2957.1444 | 2957.1426 | -0.0018 | 20.9 | |
| **no glycan** | **1.7** | 1188.5048 | 1188.5068 | 0.002 | 15.0 | |
| RVC58 | | | | | |  |
| **Glycan** | **Abundance (%)** | Theoretical mass (Da) | Observed mass (Da) | Delta (Da) | RT (min) |  |
| **G0F-Gn** | **2.5** | 2429.9593 | 2429.959 | -0.0003 | 20.3 |  |
| **G0F** | **59.0** | 2633.0387 | 2633.0535 | 0.0148 | 20.3 |  |
| **G1F** | **34.5** | 2795.0915 | 2795.0989 | 0.0074 | 20.4 |  |
| **G2F** | **4.4** | 2957.1444 | 2957.1455 | 0.0011 | 20.5 |  |
| **G2FS1** | **0.9** | 3086.187 | 3086.1914 | 0.0044 | 26.0 |  |
| **no glycan** | **1.9** | 1188.5048 | 1188.5071 | 0.0023 | 14.0 |  |

## Supplementary Videos

**Supplementary Video S1.** 3D reconstruction of the “Merge” immunofluorescence image presented in Supplementary Figure 2B, panel “RABV”.

**Supplementary Video S2.** Rotating video of the 3D view rendering panel “RABV” presented in Supplementary Figure 2B.

**Supplementary Video S3.** 3D reconstruction of the “Merge” immunofluorescence image presented in Supplementary Figure 2B, panel “RABV+RVC20”.

**Supplementary Video S4.** Rotating video of the 3D view rendering panel “RABV+RVC20” presented in Supplementary Figure 2B.

**Supplementary Video S5.** 3D reconstruction of the “Merge” immunofluorescence image presented in Supplementary Figure 2B, panel “RABV+RVC58”.

**Supplementary Video S6.** Rotating video of the 3D view rendering panel “RABV+RVC58” presented in Supplementary Figure 2B.

# Supplementary Materials and Methods

## Immunofluorescence through Opera Phenix High-Content Screening System

For the results shown in Figure 1, cells were fixed 1, 4 or 6 dpi after using 4% formaldehyde for 15 minutes at room temperature, washed with PBS. The nuclei were counterstained with Hoechst 33342. Image acquisitions of 13 fields/well (totaling 21.6 mm^2^/well) were performed on the automated confocal microscope Opera Phenix (Perkin Elmer) using the 10× objective. Data were then transferred to the Columbus Image Data Storage and Analysis System (Perkin Elmer) and the percentage of GFP-positive cells was determined. The nonspecific fluorescent signal emitted by the non-infected cells (blank) was subtracted from all the values as a background signal.

## Immunofluorescence through Leica TCS SP8 confocal microscope

For the results shown in Figure 2, SH-SY5Y cells were fixed in 4% formaldehyde for 15 minutes, washed in PBS and permeabilized by incubation in 0.1% Triton X-100 in PBS for 5 minutes. Following permeabilization, cells were incubated with a FITC-conjugated Anti-Rabies Monoclonal Globulin (Fujirebio, cat. n. 800-092) or Adsorbed Anti-Rabies nucleocapsid conjugate (Bio-rad, cat n. 3572112) and Alexa Fluor 568-conjugated goat anti-Human IgG antibody (Thermo Fisher Scientific, cat. n. A-21090) for one hour at 37° C and then washed in PBS. Nuclear staining was performed with bisbenzimide (DAPI, Sigma-Aldrich™, cat. n. D9542) for 15 minutes at room temperature. Cells were washed three times in PBS before microscope observation.

For cell membrane staining as shown in Supplementary Figure 2B, Alexa Fluor® 647 conjugated Wheat Germ Agglutinin (WGA, Thermo Fisher Scientific, cat. n. W32466) was used prior to cell permeabilization.

Images were acquired with Leica TCS SP8 confocal microscope equipped with a CCD camera using LAS AF 2.7.3.9723 software and analyzed using ImageJ.

In order to quantify RABV infection in cell cultures (Figure 2A and 2B), manual cell counting was performed using ImageJ Cell Counter plugin in fluorescent or confocal images of immunostained cells (20x objective). For fluorescent images, at least 150 cells per field in 10 representative fields were counted. For confocal images, more than 1,000 cells per field in five representative fields were counted. Percentages of infected cells were then calculated and compared among control and mAbs treated cells. Representative images were also acquired with a 63x objective (Figure 2C).

In order to quantify mAbs-bound RABV infected cells (Figure 2C), manual cell counting was performed using ImageJ Cell Counter plugin in confocal images of immunostained cells (63x objective); on average, 200 cells per field in 10 representative fields were counted. Percentages of mAbs-positive infected cells were then calculated and compared among different experimental conditions (see Material and Methods 2.4 for further details). Representative images were also acquired with a 63x objective.

3D view rendering (Supplementary Figure 2B) was performed on confocal images through Fiji-ImageJ software to analyze localization and contact of RNP complexes with cell membrane and mAbs. Z-stack images of immunostained cells were acquired with a 63x objective, then a selected portion of interest was cropped and processed for 3D visualization analysis.

## Effect of RVC20 and RVC58 monoclonal antibodies on cell viability

AlamarBlue® HS cell viability reagent (Thermo Fisher Scientific, cat. n. DAL1025) was used to evaluate the effect of RVC20 and RVC58 on the proliferation of uninfected and RABV-infected cells, according to the manufacturer's instructions. Briefly, a suspension of either cells or cells and RABV (CVS-11) at MOI 0.1 was distributed (1 ×104 cells/well) in 96-well plates and cells were grown for 24 hours before treatment with mAbs. RVC20 or RVC58 were added at 0.3, 0.6 or 1.2 µg/mL. At 2, 3 or 4 dpi, 10 μl of alamarBlue were added to each well and cells were incubated for 3 hours at 37°C. The absorbance of the samples at 570 nm was measured with a Tecan Sunrise™ absorbance microplate reader, using 600 nm as a reference wavelength. The percentage of cell viability was calculated using uninfected and untreated cells as control sample.

## ELISA for human IgG quantification in the Syrian hamsters’ central nervous system

Quantification of RVC20 and RVC58 in CVS-11-infected Syrian hamsters brains was assessed as previously described (1,2), with ELISA plates coated with a 1:10 dilution of animals central nervous system (CNS). Plates were read at 405 nm and the concentration of antibodies was determined by interpolating absorbance results for each sample with a standard curve, built with serial dilutions of RVC20 and RVC58.

## mAbs neutralization ability (Rapid Fluorescent Foci Inhibition Test)

We assessed the neutralization ability of all the tested antibodies using a modified Rapid Fluorescent Foci Inhibition Test (RFFIT) (3), recommended as reference technique by WHO (4), using a standard dose of RABV (CVS-11) as challenge virus. mAbs were diluted on a three-fold dilution basis in culture medium according to the available volume, analysed using BSR CL13 cells and commercial adsorbed Anti-Rabies nucleocapsid conjugate (Bio-rad, cat n. 3572112). Antibody titers were calculated through the Reed-Muench method and expressed as IC50 ng/mL (5), considering as seropositive the sample that inhibited viral growth at 50% at the starting dilution.

## Molecular docking and molecular dynamics

Modelled RVC58 was docked to RABV-G strain CSV-11 using HADDOCK 2.4 webserver. To improve docking likelihood the algorithm was constrained by forcing attraction between the RVC58 CDR region (residues: 23-36; 52-58; 91-100; 226-232; 252-257; 299-313) and the RABV-G antigenic site III (residues: 330-338). Moreover, no repulsion between residues was forced and all other settings were left to default. The most populated cluster was considered the best pose and underwent further analyses. Docked models underwent a small molecular dynamics (MD) simulation to minimize energy and improve model quality. Specifically, MD was performed with Gromacs 2022.3 (6,7) using the Charmm36-jul2021 force field (8,9). The models were solvated with the TIP3P water model in a rectangular box with a minimum distance of 1 nm between the protein complex and the border. 0.15 M of NaCl was added to simulate a realistic ionic strength. System energy was minimized by 5000 steps of steepest descent energy minimization, with a tolerance of 1000 kJ mol^-1^ nm^-1^. Subsequently, a 200 ps NVT MD simulation was used to heat the system from 0 to 310 K and equilibrated to 1 atm during a 1 ns NPT simulation. Positional restraints were set to 1000 kJ mol^-1^ nM^-2^ on all atoms for the energy minimization and equilibration steps; however, they were removed for the 1 µs production run. The V-rescale thermostat was used to equilibrate the temperature, whereas the C-rescale barostat was used to control the pressure (10,11). Newton’s equation of motion was integrated using a leapfrog algorithm with a 2 fs time step. The particle mesh Ewald (PME) method was used to compute the long-range electrostatic forces (12). Rotational and translational motions of the system were removed, and all bonds were constrained with the LINCS algorithm. Umbrella sampling was applied to estimate the affinity of antibody fragments to RABV-G protein. In such MD simulations, a protein complex is forced to dissociate to determine the variation of the free energy of the bound and unbound state. Dissociation (or association) of protein complexes occur in a much higher timescale than the one that can be sampled in a traditional equilibrium MD simulation. Therefore, a preliminary pulling simulation is performed to dissociate the complex. Specifically, an external pulling force is applied to one of the members of the complex to force the system to explore the unbound state in a shorter timeframe. However, the resulting dissociation trajectory is not suitable per se to infer ∆G_bind_ (13–15). Therefore, snapshots at increasing center of mass (COM) distances between the two proteins are extracted and used as starting configurations for independent MD simulations (sampling windows), with the aim of generating an ensemble of structures along the dissociation direction (reaction coordinate). The potential of mean force (PMF; i.e., the free energy surface along the reaction coordinate) can be calculated as a function of the reaction coordinate by reassembling adjacent sampling windows by the weighted histogram analysis method (WHAM) and the ∆G can then be computed as the difference between the highest and lowest point of the PMF (16,17). Pulling simulations were performed with Gromacs 2022.3. The Charmm36-jul2021 force field was used to parametrize the proteins and water was simulated with a TIP3 model. Protein complexes were put in tetrahedral boxes sized so that the box dimension along the pulling coordinate was more than twice the maximum distance between the center of mass. The system was solvated and 0.15 M NaCl was added to simulate physiological ionic strength and neutralize the system. Initial energy minimization, NVT, and NPT equilibrations were performed as previously described. The final states of the NPT simulation were used as input to perform a fast-pulling simulation (FPL) of single-chain variable fragments (scFvs) from RABVG. During such FPL simulation, the scFv was forced to dissociate from RABVG applying an external harmonic force along the reaction coordinate. Specifically, the cantilever spring constant was chosen to be k = 1000 kJ mol^−1^ nM^−2^ and the pulling velocity was set to 0.01 nm ps^-1^. Applications of slower pulling rates and forces resulted in the production of nearly identical trajectories and similar force vs time curves, thus the faster pulling rate was applied to all systems in order to hasten data collection while still preserving the reliability of the result. The FPL simulation was carried on for 500 ps, so that the center of mass (COM) of the two proteins was separated by a total of 5 nm at the end of the simulation. Windows at approximately 0.2 nm from each other were selected for independent MD simulations. Specifically, each window was equilibrated in a 200 ps NPT simulation followed by a 5 ns production run with the settings described in the previous section. Results were analyzed with the WHAM method using 50 bins and 200 rounds of bootstrapping analysis (18).

## Glycan profiling of RVC20 and RVC58 mAbs

Liquid chromatography- mass spectrometry **(**LC-MS) peptide map was used to check the glycan profiling on RVC20 and RVC58. Protein was reduced (dithiothreitol), denatured (6M guanidine hydrochloride), alkylated (Iodoacetamide), and buffer exchanged (Zeba spin desalting column) before trypsin digestion. 20 µg of digested peptide was analyzed on the LC-MS system (Thermo Vanquish UPLC and Q Exactive Plus Orbitrap MS) to acquire both MS1 and MS2 data under HCD fragmentation. Peptide mapping data was analyzed by Biopharma Finder 3.2. The glycopeptides were confirmed by their accurate mass and LC retention time.

# Supplementary References

1. De Benedictis P, Minola A, Rota Nodari E, Aiello R, Zecchin B, Salomoni A, Foglierini M, Agatic G, Vanzetta F, Lavenir R, et al. Development of broad‐spectrum human monoclonal antibodies for rabies post‐exposure prophylaxis. *EMBO Mol Med* (2016) 8:407–421. doi: 10.15252/emmm.201505986

2. Piccoli L, Campo I, Fregni CS, Rodriguez BMF, Minola A, Sallusto F, Luisetti M, Corti D, Lanzavecchia A. Neutralization and clearance of GM-CSF by autoantibodies in pulmonary alveolar proteinosis. *Nat Commun* (2015) 6: doi: 10.1038/ncomms8375

3. Warrell MJ, Riddell A, Yu LM, Phipps J, Diggle L, Bourhy H, Deeks JJ, Fooks AR, Audry L, Brookes SM, et al. A simplified 4-site economical intradermal post-exposure rabies vaccine regimen: A randomised controlled comparison with standard methods. *PLoS Negl Trop Dis* (2008) 2:1–9. doi: 10.1371/journal.pntd.0000224

4. World Health Organization. *Laboratory techniques in rabies. Fifth edition, volume 1*. Rupprecht CE, Fooks; AR, Abela-Ridder B, editors. Geneva (2018). 217 p.

5. Reed LJ, Muench H. A simple method of estimating fifty per cent endpoints. *Am J Epidemiol* (1938) 27:493–497. doi: https://doi.org/10.1093/oxfordjournals.aje.a118408

6. Berendsen HJC, van der Spoel D, van Drunen R. GROMACS: A message-passing parallel molecular dynamics implementation. *Comput Phys Commun* (1995) 91:43–56. doi: 10.1016/0010-4655(95)00042-E

7. Abraham MJ, Murtola T, Schulz R, Páll S, Smith JC, Hess B, Lindah E. Gromacs: High performance molecular simulations through multi-level parallelism from laptops to supercomputers. *SoftwareX* (2015) 1–2:19–25. doi: 10.1016/j.softx.2015.06.001

8. Soteras Gutiérrez I, Lin FY, Vanommeslaeghe K, Lemkul JA, Armacost KA, Brooks CL, MacKerell AD. Parametrization of halogen bonds in the CHARMM general force field: Improved treatment of ligand–protein interactions. *Bioorganic Med Chem* (2016) 24:4812–4825. doi: 10.1016/j.bmc.2016.06.034

9. Vanommeslaeghe K, Hatcher E, Acharya C, Kundu S, Zhong S, Shim J, Darian E, Guvench O, Lopes P, Vorobyov I, et al. CHARMM General Force Field: A Force Field for Drug-Like Molecules Compatible with the CHARMM All-Atom Additive Biological Force Fields. *J Comput Chem* (2009) 31: doi: 10.1002/jcc

10. Bussi G, Donadio D, Parrinello M. Canonical sampling through velocity rescaling. *J Chem Phys* (2007) 126:014101. doi: 10.1063/1.2408420

11. Bernetti M, Bussi G. Pressure control using stochastic cell rescaling. *J Chem Phys* (2020) 153: doi: 10.1063/5.0020514

12. Darden T, York D, Pedersen L. Particle mesh Ewald: An N⋅log(N) method for Ewald sums in large systems. *J Chem Phys* (1993) 98:10089. doi: 10.1063/1.464397

13. Patey GN, Valleau JP. The free energy of spheres with dipoles: Monte Carlo with multistage sampling. *Chem Phys Lett* (1973) 21:297–300. doi: 10.1016/0009-2614(73)80139-3

14. Torrie GM, Valleau JP. Monte Carlo free energy estimates using non-Boltzmann sampling: Application to the sub-critical Lennard-Jones fluid. *Chem Phys Lett* (1974) 28:578–581. doi: 10.1016/0009-2614(74)80109-0

15. Torrie GM, Valleau JP. Nonphysical sampling distributions in Monte Carlo free-energy estimation: Umbrella sampling. *J Comput Phys* (1977) 23:187–199. doi: 10.1016/0021-9991(77)90121-8

16. Mills M, Andricioaei I. An experimentally guided umbrella sampling protocol for biomolecules. *J Chem Phys* (2008) 129:114101. doi: 10.1063/1.2976440

17. Lemkul JA, Bevan DR. Assessing the stability of Alzheimer’s amyloid protofibrils using molecular dynamics. *J Phys Chem B* (2010) 114:1652–1660. doi: 10.1021/JP9110794/SUPPL_FILE/JP9110794_SI_001.PDF

18. Kumar S, Rosenberg JM, Bouzida D, Swendsen RH, Kollman PA. THE weighted histogram analysis method for free-energy calculations on biomolecules. I. The method. *J Comput Chem* (1992) 13:1011–1021. doi: 10.1002/JCC.540130812
